# BDNF-dependent modulation of axonal transport is selectively impaired in ALS

**DOI:** 10.1186/s40478-022-01418-4

**Published:** 2022-08-22

**Authors:** Andrew P. Tosolini, James N. Sleigh, Sunaina Surana, Elena R. Rhymes, Stephen D. Cahalan, Giampietro Schiavo

**Affiliations:** 1 Department of Neuromuscular Diseases, Queen Square Institute of Neurology, University College London, London, WC1N 3BG UK; 2 UCL Queen Square Motor Neuron Disease Centre, University College London, London, WC1N 3BG UK; 3 UK Dementia Research Institute, University College London, London, WC1E 6BT UK; 4 Comparative Neuromuscular Disease Laboratory, Department of Clinical Sciences and Services, Royal Veterinary College, University of London, London, NW1 0TU UK

**Keywords:** Axonal transport, Amyotrophic lateral sclerosis, Motor neuron, BDNF, TrkB, p75^NTR^

## Abstract

Axonal transport ensures long-range delivery of essential cargoes between proximal and distal compartments, and is needed for neuronal development, function, and survival. Deficits in axonal transport have been detected at pre-symptomatic stages in the SOD1^G93A^ and TDP-43^M337V^ mouse models of amyotrophic lateral sclerosis (ALS), suggesting that impairments in this critical process are fundamental for disease pathogenesis. Strikingly, in ALS, fast motor neurons (FMNs) degenerate first whereas slow motor neurons (SMNs) are more resistant, and this is a currently unexplained phenomenon. The main aim of this investigation was to determine the effects of brain-derived neurotrophic factor (BDNF) on in vivo axonal transport in different α-motor neuron (MN) subtypes in wild-type (WT) and SOD1^G93A^ mice. We report that despite displaying similar basal transport speeds, stimulation of wild-type MNs with BDNF enhances in vivo trafficking of signalling endosomes specifically in FMNs. This BDNF-mediated enhancement of transport was also observed in primary ventral horn neuronal cultures. However, FMNs display selective impairment of axonal transport in vivo in symptomatic SOD1^G93A^ mice*,* and are refractory to BDNF stimulation, a phenotype that was also observed in primary embryonic SOD1^G93A^ neurons. Furthermore, symptomatic SOD1^G93A^ mice display upregulation of the classical non-pro-survival truncated TrkB and p75^NTR^ receptors in muscles, sciatic nerves, and Schwann cells. Altogether, these data indicate that cell- and non-cell autonomous BDNF signalling is impaired in SOD1^G93A^ MNs, thus identifying a new key deficit in ALS.

**Supplementary Information:**

The online version contains supplementary material available at 10.1186/s40478-022-01418-4.

## Introduction

Amyotrophic lateral sclerosis (ALS) is a progressive neurodegenerative disease primarily affecting motor neurons (MNs), leading to muscle atrophy, paralysis and ultimately death due to respiratory failure. Although only a small proportion of ALS-causing mutations are found in genes encoding components of the axonal transport machinery (e.g., *KIF5A, DCTN1, ANXA11*), altered axonal transport is a common pathological feature downstream of many ALS-causing mutations [1, 2]. Axonal transport maintains neuronal homeostasis by ensuring the long-range delivery of several cargoes, including cytoskeletal components, organelles, signalling molecules and RNA between proximal and distal neuronal compartments [3]. As a result, perturbations in axonal transport have severe consequences for neuronal homeostasis and function [4]. Indeed, we have previously demonstrated that deficits in in vivo axonal transport occur pre-symptomatically (i.e., before MN loss) across diverse ALS mice [5–9].

α-MNs are defined by the type of skeletal muscle fibre they innervate, and can be sub-classified according to their firing pattern into fast (FMNs) and slow (SMNs) MNs, each with distinct anatomical, metabolic, and functional properties [10–13], as well as diverse transcriptional profiles [14]. FMNs innervate type-IIb and -IIx fast-twitch fatigable and type IIa fast-twitch fatigue-resistant muscle fibres to execute fine motor control, whereas SMNs innervate type I slow-twitch fatigue-resistant muscle fibres to exert postural control [11]. Strikingly, FMNs are more susceptible to ALS pathology, whereas SMNs are predominantly resistant [13]. Preferential FMN vulnerability has been observed in SOD1 [15–17], TDP-43 [18], FUS [19] and C9ORF72 [20] mutant mice, with limb-onset ALS accounting for ~ 70% of human pathology [21], suggesting that preferential FMN vulnerability in ALS is conserved across species.

The exclusively FMN-innervated tibialis anterior (TA) muscle [22–24] undergoes pathological changes early in disease in ALS mice [15–17, 19], with neuromuscular junction (NMJ) denervation occurring before MN loss [25], and pathology in TA is observed in ALS patients [26]. In contrast, the predominantly SMN-innervated soleus muscle is more resistant to pathology [19, 23–25]. ALS induces a fast-to-slow muscle fibre type switch in the TA, with a significant reduction in type IIb fibres and a concomitant increase in type IIa/IIx fibres [23, 24, 27]. This precedes NMJ denervation and has been, at least in part, attributed to a metabolic switch in fast-twitch glycolytic muscles [28, 29]. Intriguingly, this fast-to-slow muscle fibre switching is also observed in mice with muscle-specific ablation of brain-derived neurotrophic factor (BDNF), with phenotypes including reduced type IIb muscle fibres, motor endplate size, and expression of muscle-specific glycolytic genes, with concomitant increases in the amount of type IIx muscle fibres [22]. Furthermore, neurotrophic factors have been shown to regulate muscle and MN subtype identities [30]. Indeed, BDNF mediates fast glycolytic fibre types [22], neurturin regulates slow-twitch motor unit development [31], whilst γ-MNs require muscle spindle-derived glial cell-derived neurotrophic factor (GDNF) for postnatal survival [32].

The neurotrophin BDNF controls the development and maintenance of neurons through binding to TrkB and p75^NTR^ receptors. TrkB exists as three differentially spliced isoforms, namely the full-length TrkB receptor (TrkB.FL) and two shorter, kinase-deficient truncated isoforms, TrkB.T1 and TrkB.T2 [33]. The cytoplasmic tyrosine kinase domain present in TrkB.FL is fundamental for pro-survival signalling via ERK1/2, Akt and PLC-γ controlled pathways [34]. However, activation of these pathways is dampened by TrkB.T1 and TrkB.T2, which lack the essential kinase domain and sequester synaptic BDNF [35]. The physiological roles of p75^NTR^ are equally complex [36], with higher affinity for pro-neurotrophins and a primary role in controlling neuronal apoptosis during development, whilst modulating neurotransmitter availability and NMJ organisation in the mature nervous system [37]. BDNF binding triggers TrkB.FL, TrkB.T1 and p75^NTR^ homo- and/or hetero-dimerisation [36], and each complex elicits distinct signalling outputs (e.g., TrkB.FL-TrkB.T1 heterodimers inhibit TrkB.FL autophosphorylation) [35, 38]. Importantly, ALS patient spinal cords display abnormality in TrkB-mediated intracellular signalling [39], as well as increased p75^NTR^ expression [40].

Despite in-depth knowledge of BDNF biology [34], the physiological landscape of BDNF signalling at the NMJ, as well as its possible perturbation in ALS, are currently  less known. BDNF regulates both the pre- and post-synaptic components of the neuromuscular synapse [22], and is secreted by skeletal muscles during contraction [38]. Internalised BDNF-receptor complexes induce both local [34] and long-distance signalling [41]. The former controls local translation at nerve terminals [42], whilst the latter is driven by sorting of activated Trk receptors [43] to signalling endosomes, which undergo fast retrograde axonal transport to the soma [44], with signalling endosome flux dependent on TrkB activation [45]. Hence, understanding the regulation of BDNF-signalling in MN subtypes can provide novel clues regarding selective MN vulnerability in ALS.

Here, we assessed axonal transport dynamics of signalling endosomes in axons of different α-MN subtypes in wild-type (WT) and SOD1^G93A^ mice in vivo. We find that BDNF stimulation promotes faster retrograde transport speeds of signalling endosomes in WT FMNs, but not in SMNs, as well as in embryonic primary ventral horn neurons. In SOD1^G93A^ mice, transport is preferentially impaired in FMNs innervating TA, which become refractory to BDNF stimulation, a phenotype we also observed in cultured SOD1^G93A^ embryonic primary ventral horn neurons. In addition, we show that truncated TrkB isoforms and p75^NTR^ levels are upregulated in muscles, sciatic nerves and Schwann cells of SOD1^G93A^ mice, thus identifying cell- and non-cell-autonomous dysregulation of BDNF signalling in ALS pathology.

## Materials and methods

### Animals

Mouse experiments were performed under license from the United Kingdom Home Office in accordance with the Animals (Scientific Procedures) Act (1986) and approved by the UCL Queen Square Institute of Neurology Ethics Committee. Mice were housed in individually ventilated cages in a controlled temperature/humidity environment and maintained on a 12 h light/dark cycle with ad libitum access to food and water. Transgenic mice carrying the mutant SOD1^G93A^ transgene (TgN[SOD1-G93A]1Gur) were obtained from the Jackson Laboratory [46]. Colonies were maintained by breeding male heterozygous carriers with female (C57BL/6 × SJL) F1 hybrids. Mice were genotyped for the human SOD1 transgene using DNA extracted from ear notches and primers as previously described [5–7]. Female and male SOD1^G93A^ mice display distinct patterns of disease, including differences in disease onset, progression and survival [47]. Therefore, only female hemizygous transgenic mice carrying the human SOD1^G93A^ transgene (hereafter referred to as SOD1^G93A^) and WT littermates were used, allowing comparisons with our previous studies [5–8]. All experimental groups contained age-matched WT and SOD1^G93A^ littermates to minimise the potential impact of differing oestrous cycles. For axonal transport, female WT mice had a mean age of 85.13 ± 14.97 days; we did not assess WT axonal transport separately at P73 and P94 as we have previously shown that there are no significant differences in transport between 1 and 18 months in WT mice [9, 48]. Female SOD1^G93A^ postnatal day 73 (P73) mice had a mean age of 72.74 ± 0.67 and P94 mice had a mean age of 93.70 ± 0.46.

### In vivo axonal transport

**Fig. 1:**
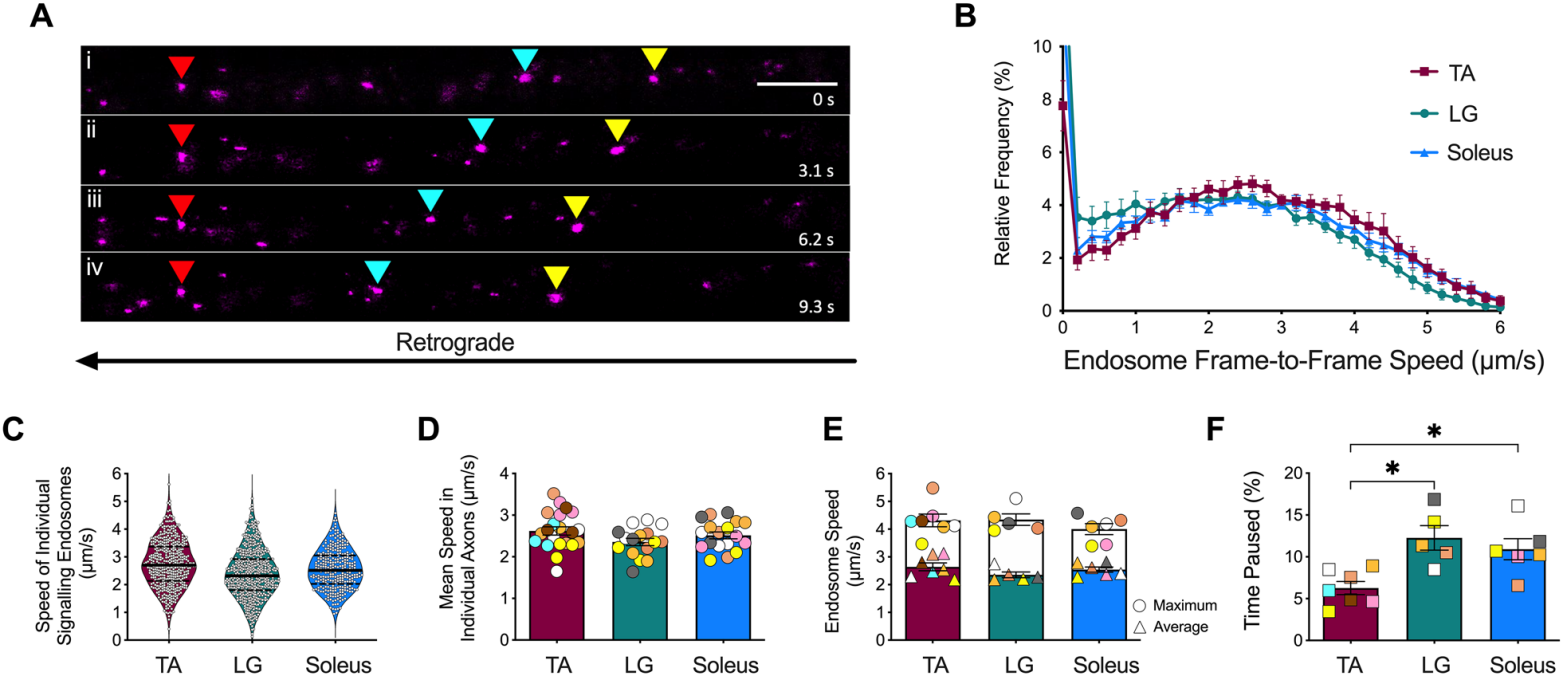
In vivo axonal transport dynamics of signalling endosomes are similar in motor neurons innervating fast and slow muscles in wild-type mice. **A** Time-lapse images of retrogradely transported (i.e., right-to-left), H_C_T-555-containing signalling endosomes from a single sciatic nerve axon. Yellow and cyan arrowheads indicate retrogradely moving signalling endosomes, while red arrowheads point to a paused endosome. Frame rate = 3.1 frame/s; scale bar = 10 µm. **B** Endosome frame-to-frame speed distribution curves in motor axons innervating tibialis anterior (TA), lateral gastrocnemius (LG) and soleus muscles in basal conditions. **C** Speed of individual H_C_T-555-positive signalling endosomes (white circles) in motor axons innervating TA, LG, and soleus. Black line represents the median and the dashed lines represent the upper and lower quartiles. No statistical comparisons were performed on these datasets due to overpowering. **D** Mean speeds of H_C_T-555-positive signalling endosomes per individual motor neuron axons innervating TA, LG, and soleus (*p =* 0.135, one-way ANOVA, n = 16–21). **E** Average (triangles) and maximum (circles) endosome speeds per animal in motor axons innervating TA, LG, and soleus (average: *p =* 0.193; maximum: *p =* 0.715; one-way ANOVA, n = 5–7). **F** Relative percentage of time signalling endosomes paused per animal in axons innervating the TA, LG, and soleus (**p = 0.002, one-way ANOVA, n = 5–7). The colour coding of individual datapoints is consistent within the muscle type and reflects the same animal in Fig. 1D, 1E and 1F. *p < 0.05, Kruskal–Wallis multiple comparisons test. See also Additional file 2: Table S1

Signalling endosomes were visualised in vivo by injecting the fluorescent atoxic binding fragment of tetanus neurotoxin (H_C_T-555), as previously described [49, 50]. Briefly, H_C_T (residues 875–1315) fused to an improved cysteine-rich region was expressed in bacteria as a glutathione-S-transferase fusion protein [51], cleaved and subsequently labelled with AlexaFluor555 C_2_ maleimide (Thermo Fisher Scientific, A-20346). 5–7.5 µg of H_C_T-555 alone, or in combination with 25 ng of human recombinant BDNF (Peprotech, 450–02) or 25 ng of human recombinant GDNF (Peprotech, 450–10) (pre-mixed with phosphate buffered saline) were injected into single muscles. Briefly, after anaesthesia was initiated and maintained using isoflurane, the fur on the ventral and/or dorsal lower leg was shaved, and mice were placed on a heat-pad for the duration of the surgery. A small incision was made using iris spring scissors on the ventral surface below the patella for TA, or on the lateral aspect of the dorsal surface below the popliteal fossa for lateral head of gastrocnemius (LG). Injections were performed as a single injection targeting the motor end plate region [52] in a volume of ~ 3.5 µl using a 701 N Hamilton® syringe (Merck, 20,779) for TA and LG. For soleus injections, a vertical incision was made on the skin covering the lateral surface of lower hindlimb between the patella and tarsus to expose the underlying musculature. Subsequent vertical incisions were carefully made laterally along the connective tissue between LG and TA, and the deeper soleus muscle was exposed using forceps. 1 µl injections were performed into soleus using pulled graduated, glass micropipettes (Drummond Scientific, 5-000-1001-X10), as previously described [53]. The overlying skin was then sutured, and mice were monitored for up to 1 h. 4–8 h later, mice were re-anaesthetised with isoflurane, and the skin covering the entire lateral surface of the injected hindlimb was removed, along with the biceps femoris muscle to expose the underlying sciatic nerve. The connective tissue underneath the sciatic nerve was loosened using curved forceps to enable the placement of a small piece of parafilm aiding the subsequent imaging. The anaesthetised mouse was then transferred to an inverted LSM780 confocal microscope (Zeiss) enclosed within an environmental chamber maintained at 37 °C. Using a 40x, 1.3 NA DIC Plan-Apochromat oil- immersion objective (Zeiss), axons containing retrogradely mobile H_C_T-555-positive signalling endosomes were imaged every 0.3–0.4 s using an 80 × digital zoom (1024 × 1024, < 1% laser power) (Fig. 1A, Additional file 1: Video S1); movies of three to five axons per animal were acquired. All imaging was concluded within 1 h of initiating anaesthesia.

### In vivo axonal transport analysis

Confocal “.czi” images were opened in FIJI/ImageJ (http://rsb.info.nih.gov/ij/), converted to “.tiff” and transport dynamics were then assessed semi-automatically (i.e., automated spot detection, followed by manual linking (see Additional file 1: Video S1)) using the TrackMate plugin [54]. Indeed, as determined by several parameters such as fluorescence intensity and diameter, the TrackMate automated spot detection method encloses all cargoes that fit the criteria within purple circles (Additional file 1: Video S1B). Endosomes selected for transport analysis were then manually connected across multiple adjacent frames (Additional file 1: Video S1C). This method provides single frame-to-frame velocities, which were then averaged across the entire run to give an average speed for each tracked endosome (as represented by an individual data point in Fig. 1C). Kymographs (Additional file 2: Fig. S2) were generated using FIJI/ImageJ to highlight axonal transport phenotypes, but were not used to assess axonal transport dynamics. Only thicker axons were selected for tracking [9]. Signalling endosomes with the following criteria were analysed: (1) organelles were tracked for a minimum of 10 and a maximum of 100 consecutive frames (i.e., ~ 3–40 s), including pauses. Terminal pausing carriers, which we defined by the absence of movement in ≥ 10 consecutive frames, were excluded; (2) for every individual axon, 15–40 signalling endosomes were tracked across at least 1000 frames (i.e., ~ 5–10 min); and (3) signalling endosome data representing an individual animal were comprised from at least three separate motor axons. The individual datapoints obtained from each experimental group can be found in Additional file 2: Table S1. Relative frequency curves were generated to display the relative frame-to-frame movements of all signalling endosomes per animal (e.g., Fig. 1B). For all mice included in the analysis, the speeds of all individual endosomes were plotted (e.g., Fig. 1C), the mean speeds of all endosomes per individual axon were averaged (e.g., Fig. 1D), and finally, the mean speed of all endosomes per animal was also averaged (e.g., triangles in Fig. 1E). Importantly, the mean values across all analyses (e.g., Fig. 1C–E) were similar. For example, for WT soleus transport, the mean speed of all individual endosomes was 2.55 µm/s (Fig. 1C), the mean endosome speed per axon was 2.51 µm/s (Fig. 1D) and the mean endosome speed per animal was 2.54 µm/s (Fig. 1E). Owing to statistical overpowering of individual endosome speed data, statistical tests were only performed on the mean endosome speeds per axon (Fig. 1D) and per animal (Fig. 1E). The fastest individual endosome speed per animal was considered as the maximum speed (e.g., represented by circles in Fig. 1E). A pause was defined by an endosome that moved less than 0.1 µm between consecutive frames, and the time paused (%) is determined by the number of pauses divided by the total number of frame-to-frame movements assessed per animal (e.g., Fig. 1F).

### In vitro axonal transport

Mixed ventral horn cultures were prepared as previously described [6–8]. Briefly, ventral horns from E11.5–13.5 WT and SOD1^G93A^ mice were dissociated, centrifuged at 380 × g for 5 min, seeded into two-chambered microfluidic devices (Fig. 3A) [7], and maintained in motor neuron media (Neurobasal (Gibco) with 2% B27 (Gibco), 2% heat-inactivated horse serum, 1% Glutamax (Invitrogen), 24.8 µM β-mercaptoethanol, 10 ng/ml ciliary neurotrophic factor (Peprotech, 450–13), 0.1 ng/ml GDNF (Peprotech, 450–10), 1 ng/ml BDNF (Peprotech, 450–02) and 1 × penicillin streptomycin (Thermo Fisher; 15140122)) at 37 °C and 5% CO_2_. After 6 days in vitro (DIV6), 30 nM H_C_T-555 and ± 50 ng/ml of BDNF was added to existing media for 45 min, then all media was replaced with fresh MN media containing 20 mM HEPES–NaOH (pH 7.4) ± 50 ng/ml of BDNF for time-lapse microscopy. Live imaging was performed on an inverted LSM780 confocal microscope at 37 °C using a 40x, 1.3 NA DIC Plan-Apochromat oil- immersion objective (Zeiss). Videos were taken at 2 frames/s for > 2.5 min. Videos were manually tracked using TrackMate [54] to determine endosome track dynamics (Fig. 3). The breakdown of each experimental group can be found in Additional file 2: Table S2.

### In vitro TrkB and p75^NTR^ western blot analysis

Mixed ventral horn cultures from E11.5–13.5 WT and SOD1^G93A^ mouse spinal cords were prepared as above, and plated in MN media in a 12-well plate coated with poly-ornithine (1.5 mg/ml) and laminin (3 µg/ml). On DIV 6–7, each well was washed once in ice-cold PBS and lysates were prepared in RIPA buffer (50 mM Tris–HCl pH 7.5, 150 mM NaCl, 1% NP-40, 0.5% sodium deoxycholate, 0.1% SDS, 1 mM EDTA, 1 mM EGTA) with freshly added Halt™ protease and phosphatase inhibitor cocktail (1:100, Thermo Fisher), and incubated on ice for 30 min. Lysates were spun at 14800 rpm at 4 °C for 15 min, the supernatant was then resuspended in 4 × Laemmli sample buffer (15% SDS, 312.5 mM Tris–HCl pH 6.8, 50% glycerol, 10% β-mercaptoethanol, 0.1% bromophenol blue) and loaded into 4–15% Mini-PROTEAN® TGX Stain-Free™ protein gels (Bio-Rad). Western blotting was then performed using standard protocols. The primary antibodies used were TrkB (R&D Systems, AF1494, 1:500) and p75^NTR^ (Biolegend, 239701, 1:500) (see Additional file 2: Table S3). Densitometry was performed using the bands at ~ 140 kDa for TrkB.FL (Additional file 2: Fig. S2Ai), ~ 75–100 kDa for truncated TrkB isoforms (Additional file 2: Fig. S2Ai) and ~ 70–85 kDa for p75^NTR^ (Additional file 2: Fig. S2Aii). Post-immunoblotting Coomassie staining [55] (Additional file 2: Fig. S2Aiii) between 60–150 kDa and 60–100 kDa was used to estimate total protein for TrkB (full-length and truncated isoforms) and p75^NTR^, respectively. This total protein load was used as an internal reference to accurately quantitate protein levels, and data were then normalised using the sum of all data points per replicate [56].

### Axon diameters

The axon diameters were measured following protocols established in ChAT.eGFP mice [9], using the same videos in the axonal transport analyses. Briefly, axon diameters were assessed by measuring the upper and lower positions of moving H_C_T-555 signalling endosomes from consecutive frames in unprocessed (i.e., not dissected, fixed, or sectioned), anatomically connected individual axons. A minimum of 10 positions were averaged for a single axon, and the mean axon diameters per animal were determined by averaging all axons from that animal (n ≥ 3 axons per animal). Similar to the in vivo transport experiments, this quantification is reliant upon intact NMJs, which can internalise H_C_T-555; hence, we cannot extrapolate diameters from denervated axons.

### Muscle BDNF, TrkB and p75^NTR^ western blot analysis

P73 (n = 5) and P94 (n = 5) WT and SOD1^G93A^ mice were culled, and fresh TA and soleus muscles were immediately dissected, snap frozen in liquid nitrogen and stored at -80 °C. Protein extraction from frozen muscles was achieved by mechanically disrupting the tissue using a scalpel, followed by immersion in RIPA buffer containing freshly added Halt^TM^ protease and phosphatase inhibitor cocktail for 15 min on ice, and then homogenised on ice using an electrical homogeniser. Lysates were incubated at 4 °C with mild agitation for 2 h, after which they were centrifuged at 21000 g for 30 min at 4 °C. 20 μl of supernatant (~ 25–40 μg of protein) was treated with 6.5% trichloroacetic acid and the resulting pellet was washed with acetone. Proteins were resuspended in 1 × Laemmli buffer and loaded on 4–12% Bis–Tris polyacrylamide gels prior to western blotting. The primary antibodies used were against BDNF (Alomone ANT-010), TrkB (Millipore, 07–225) and p75^NTR^ (Biolegend, 839701) (all 1:1000; see Additional file 2: Table S3). Densitometry was performed on the bands at ~ 20 kDa for BDNF (Fig. 5A), ~ 140 kDa for TrkB.FL (Fig. 5B), ~ 75–100 kDa for truncated TrkB (Fig. 5B) and ~ 70–85 kDa for p75^NTR^ (Fig. 5C). As the steady-state levels of standard housekeeping proteins, such as GAPDH and β-actin, differ between muscle types, and can be affected by age, sex, and pathology [57, 58], post-immunoblotting Coomassie staining [55] between 10–25 kDa,  70-150 kDa and 60-100 kDa was used to assess relative levels of BDNF, TrkB (full-length and truncated isoforms), and p75^NTR^, respectively. The total protein load was used as an internal reference to accurately quantify relative protein levels, and data were then normalised using the sum of all data points in a replicate [56]. P73 and P94 WT and SOD1^G93A^ data points were combined as there were no timepoint-specific differences (data not shown).

### Muscle immunohistochemistry (IHC)

P73 (n = 3) and P94 (n = 3) WT and SOD1^G93A^ mice were culled, and TA and soleus muscles were immediately dissected and post-fixed in 4% paraformaldehyde (PFA) for 15–60 min. Muscle fibres were teased apart in bundles of 1–10 fibres and stained with α-bungarotoxin (BTX; Thermo Fisher Scientific, B13423, 1:500) for 1 h. Fibres were then permeabilized with 2% Triton X-100 in PBS for 90 min, then immersed in a blocking solution containing 4% bovine serum albumin and 1% Triton X-100 in PBS for 30 min at room temperature. Primary antibodies (see Additional file 2: Table S3) against TUJ1 (Synaptic Systems, 302306, 1:50), synaptophysin (Syn; Synaptic Systems, 101006, 1:50), TrkB (Millipore, 07–225, 1:50), p75^NTR^ (Promega, G3231, 1:50) and S100 (Atlas Antibodies, AMAb91038, 1:250) immersed in blocking solution were added to the teased muscle fibres for ~ 3 d at 4 °C with mild agitation, and then washed in PBS at room temperature. Secondary antibodies (see Additional file 2: Table S4) in PBS were then applied to fibres for ~ 1 h at room temperature, followed by multiple washes in PBS and then finally mounted on SuperFrost Plus slides (VWR, 631–0108) using Mowiol. Slides were dried and imaged with a LSM780 confocal microscope using a 63 × Plan-Apochromat oil immersion objective (Zeiss). A minimum of 25 NMJs were imaged per condition, comprised of fully or partially innervated, but not denervated, NMJs. Mean fluorescence was measured using FIJI/ImageJ by applying an overlapped Syn/TUJ1-BTX mask to the TrkB or p75^NTR^ immunolabelled regions, and the mean fluorescence per animal was assessed by averaging all the individual data points. For the TrkB analysis, we assessed the WT TA (n = 6, NMJs = 182), WT soleus (n = 6, NMJs = 223), SOD1^G93A^ TA (n = 6, NMJs = 180) and SOD1^G93A^ soleus (n = 6, NMJs = 191) muscles. For the p75^NTR^ analysis, we assessed the WT TA (n = 6, NMJs = 176), WT soleus (n = 6, NMJs = 224), SOD1^G93A^ TA (n = 6, NMJs = 183) and SOD1^G93A^ soleus (n = 6, NMJs = 198) muscles. P73 and P94 WT and SOD1^G93A^ data points were combined as there were no timepoint specific differences (data not shown).

### Sciatic nerve western blot analysis

P73 (n = 5) and P94 (n = 5) WT and SOD1^G93A^ mice were culled, and sciatic nerves were immediately dissected, snap frozen in liquid nitrogen and stored at − 80 °C. Thawed sciatic nerves were then immersed in NP-40 lysis buffer (150 mM NaCl, 1% NP-40, 50 mM Tris–HCl, pH 8.0) with freshly added Halt^TM^ protease and phosphatase inhibitor cocktail (10% weight/volume) (100x, Fisher, 78442). Lysates underwent mechanical disruption using a plastic pestle before being left on ice for 0.5 h and then centrifuged for 20 min at 10,000* g*. Proteins were re-suspended in 4 × Laemmli buffer, and 40 µg/sample were loaded on 4–12% Bis–Tris polyacrylamide gels prior to western blotting. Primary antibodies (see Additional file 2: Table S3) against TrkB (Millipore, 07–225, 1:1000), p75^NTR^ (Biolegend, 839701, 1:2000), ERK1/2 (CST, 9102, 1:1000), p-ERK1/2 (CST, 9101, 1:1000), AKT (CST, 9272, 1:1000), p-AKT (CST, 9275, 1:1000) and Cofilin (Cytoskeleton, ACFL02, 1:500) were used to quantify protein levels. *N.B.* The expression of TrkB.FL, AKT and p-AKT were below detection levels. All bands were first standardised to cofilin, and then normalised by the sum of all data points in a replicate [56]. P73 and P94 WT and SOD1^G93A^ data points were combined as there were no timepoint specific differences (data not shown).

### Sciatic nerve IHC

P73 (n = 4) WT and SOD1^G93A^ mice were culled, and sciatic nerves were immediately dissected, post-fixed in a 4% PFA solution in PBS overnight at 4 °C, cryopreserved in a 30% sucrose solution in PBS for 2 d at 4 °C, and finally frozen in OCT (Agar Scientific, AGR1180). 30 µm longitudinal cryosections of sciatic nerves were directly mounted on SuperFrost Plus slides (VWR, 631–0108), and a hydrophobic barrier pen (Vector Laboratories, H-4001) was then applied to the slides surrounding the sectioned tissue. PBS rehydrated tissue was then blocked using 10% normal horse serum in 0.2% Triton X-100 in PBS for ~ 1 h and then primary antibodies (see Additional file 2: Table S3) specific for S100 (Merck, S2532, 1:200), TUJ1 (Synaptic systems, 302306, 1:500), TrkB (Millipore, 07–225, 1:250) and p75^NTR^ (Promega, G3231, 1:500) were applied overnight at room temperature. After multiple PBS washes, the secondary antibodies (see Additional file 2: Table S4) in PBS were applied for 2–3 h, followed by multiple washes and then mounted with Mowiol. Slides were dried and imaged as described above. A minimum of six sciatic nerve sections were imaged per condition. Mean fluorescence was measured by applying a TUJ1 and S100 mask to the TrkB or p75^NTR^ regions, and the mean fluorescence per animal was assessed by averaging all the individual data points.

### Statistical analyses

GraphPad Prism 9 (GraphPad Software) was used for statistical analyses. Normal distribution was first ascertained by the D’Agostino and Pearson omnibus normality test, and parametric data were statistically assessed using unpaired, two-tail *t*-tests, one-way or two-way analyses of variance (ANOVA) with Holm-Sidaks multiple comparison tests. Non-normally distributed data were analysed by a two-tailed Mann–Whitney *U* test or Kruskal–Wallis test with Dunn’s multiple comparisons test.

## Results

### In vivo axonal transport is differentially regulated in MN subtypes by BDNF

To investigate the influence of α-MN and skeletal muscle subtypes [10–12] on axonal transport dynamics in vivo, we labelled neurotrophin-containing signalling endosomes with a fluorescent atoxic tetanus neurotoxin binding fragment (H_C_T) [59, 60] to assess axonal transport dynamics in sciatic nerves of live mice [49, 50]. H_C_T is internalised into MNs upon binding to nidogens and polysialogangliosides at distal terminals [61], and is retrogradely transported in Rab7-positive signalling endosomes [44]. Using H_C_T-555, we separately targeted the FMN-innervated TA, the FMN- and SMN-innervated (i.e., mixed) LG and the predominantly SMN-innervated soleus muscles in WT mice, and after 4–8 h, we performed time-lapse intravital microscopy (Fig. 1A; Additional file 1: Video S1). Speed distribution curves (Fig. 1B), as well as the average and maximum velocities (Figs. 1C-E) indicate that endosome transport dynamics in axons innervating TA, LG and soleus are similar, albeit with less pausing in TA axons (Fig. 1F).

**Fig. 2:**
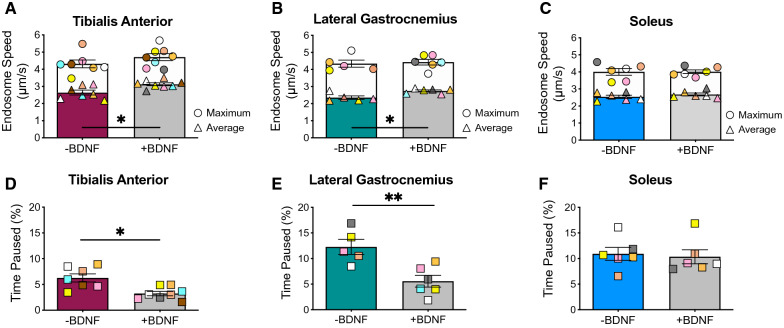
BDNF stimulation differentially impacts axonal transport of signalling endosomes in motor neurons innervating fast and slow muscles in wild-type mice. **A** Average and maximum endosome speeds upon intramuscular BDNF stimulation in motor axons innervating tibialis anterior (average: *p = 0.029; maximum: *p =* 0.281), **B** lateral gastrocnemius (average: *p = 0.017; maximum: *p =* 0.178), and **C)** soleus (average: *p =* 0.485; maximum: *p =* 0.937). **D** Time signalling endosomes paused upon BDNF stimulation in motor axons innervating tibialis anterior (*p = 0.014), **E** lateral gastrocnemius (**p = 0.009) and **F** soleus (*p =* 0.559). Data were assessed by Mann–Whitney *U* tests (n = 5–8). Means ± SEM are plotted for all graphs. The colour coding of individual datapoints is consistent within the muscle and treatment type and reflects the same animal. *p < 0.05, **p < 0.01. See also Additional file 2: Figs. S1 and Table S1

We next assessed whether peripheral stimulation with BDNF impacts signalling endosome transport dynamics, given the influence of this neurotrophin on endocytosis, endosomal flux and pro-survival signalling events [41, 45]. Co-injection of H_C_T-555 with 25 ng of recombinant BDNF increased the mean speeds of signalling endosomes in motor axons innervating the TA (Fig. 2A; Additional file 2: Fig. S1A) and LG (Fig. 2B; Additional file 2: Fig. S1B), whilst concurrently reducing their pausing (Figs. 2D-E). However, BDNF stimulation had no influence on transport in soleus motor axons (Fig. 2C, F; Additional file 2: Fig. S1C). We then tested if this response was specific for BDNF or a general feature of neurotrophic factors, by stimulating FMN axons with GDNF, which is known to activate distinct signalling cascades via RET and GFRα receptors [8, 12]. In contrast to BDNF, application of 25 ng of recombinant GDNF did not influence transport of H_C_T-555-positive signalling endosomes (Additional file 2: Fig. S1D-E). Altogether, these data indicate that FMNs and SMNs have similar axonal transport speeds under basal conditions, and that BDNF stimulation enhances axonal transport dynamics specifically in FMNs.

### Axonal transport is selectively impaired in TA-innervating axons of SOD1^G93A^ mice

**Fig. 3:**
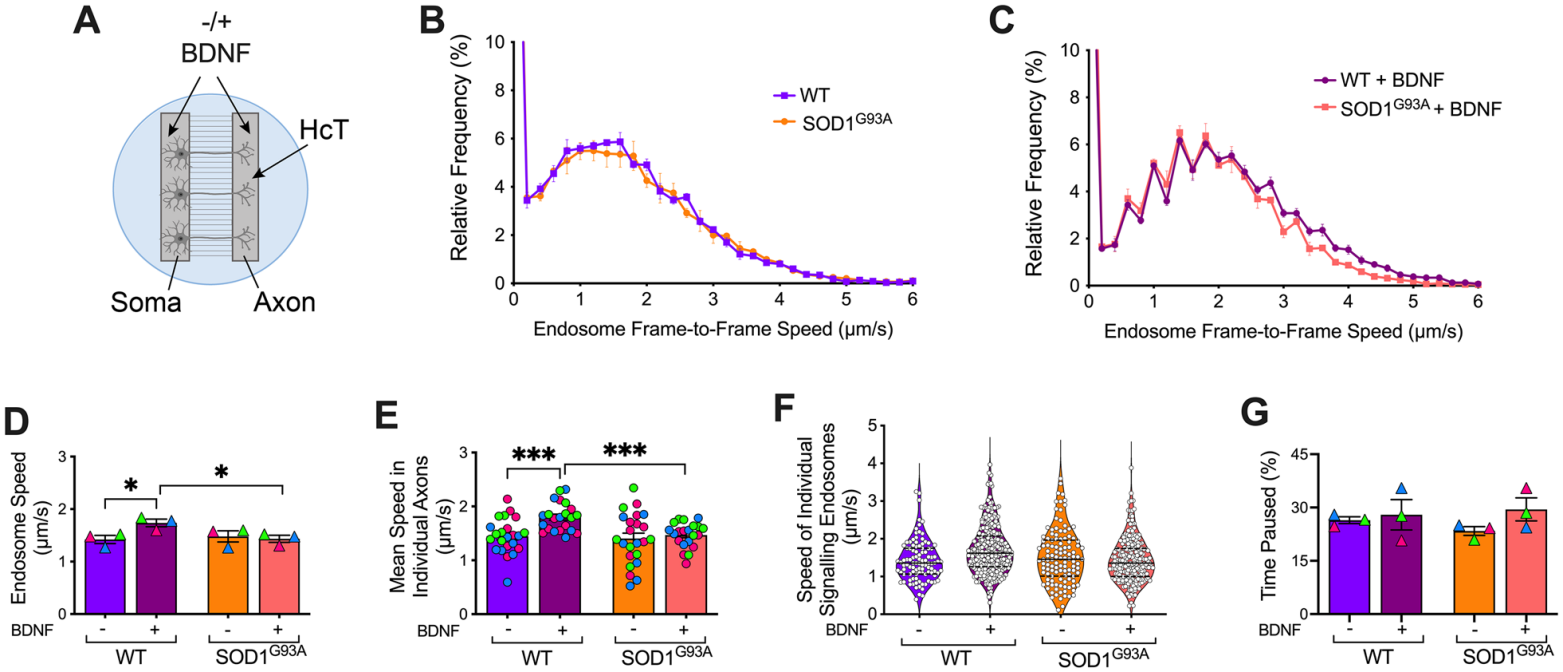
BDNF stimulation enhances axonal transport of signalling endosomes in primary embryonic ventral horn neurons from WT, but not SOD1^G93A^ mice.** A** Schematic of primary embryonic ventral horn cultures plated in microfluidic chambers (MFCs) with or without 50 ng/µl of BDNF added to both somatic and axonal compartments. **B** Speed distribution curves of signalling endosome transport in WT and SOD1^G93A^ primary ventral horn neurons in MFCs. **C** Axonal transport dynamics of the same cultures upon addition of 50 ng/ml of BDNF, with the mean endosome speeds shown in **D** (WT vs. SOD1^G93A^: *p =* 0.068; WT −/ + BDNF: *p = 0.041; SOD1^G93A^ −/ + BDNF: *p =* 0.735; WT vs. SOD1^G93A^ + BDNF: *p = 0.037). **E** Mean speeds in individual motor neuron axons (WT vs. SOD1^G93A^: *p =* 0.7308; WT −/ + BDNF: ***p < 0.001; SOD1^G93A^ -/ + BDNF: *p =* 0.576; WT vs. SOD1^G93A^ + BDNF: ***p < 0.001). **F** Speed of individual H_C_T-555-positive signalling endosomes (white circles; black lines represent the median and the dashed lines represent the upper and lower quartiles). **G** Percentage of signalling endosomes pausing (WT vs. SOD1^G93A^: *p =* 0.122; WT −/ + BDNF: *p =* 0.745; SOD1^G93A^ −/ + BDNF: *p =* 0.154; WT vs. SOD1.^G93A^ + BDNF: *p =* 0.791). Statistical analyses were performed using unpaired, two-tailed *t*-tests (n = 3 biological replicates). See also Additional file 2: Table S2

SOD1^G93A^ mice display early and persistent axonal transport deficits [5], but the precise contributions of fast and slow MNs, as well as BDNF stimulation, are currently unresolved. To fill this gap, we first assessed the effect of BDNF on in vitro axonal transport in embryonic primary ventral horn neurons in microfluidic chambers (Fig. 3A). Under basal conditions, we observed similar transport speeds between WT and SOD1^G93A^ neurons (Fig. 3B, D-F), thus supporting a neurodegenerative, rather than neurodevelopmental, transport phenotype in SOD1^G93A^ mice [5]. Moreover, TrkB.FL, truncated TrkB and p75^NTR^ levels do not differ between WT and SOD1^G93A^ cultures (Additional file 2: Fig. S2). However, application of 50 ng/ml of recombinant BDNF increased WT endosome retrograde transport speeds (without altering pausing), but had no effect in SOD1^G93A^ primary ventral horn neurons, suggestive of dysregulated BDNF signalling in SOD1^G93A^ MNs (Fig. 3C–G).

**Fig. 4:**
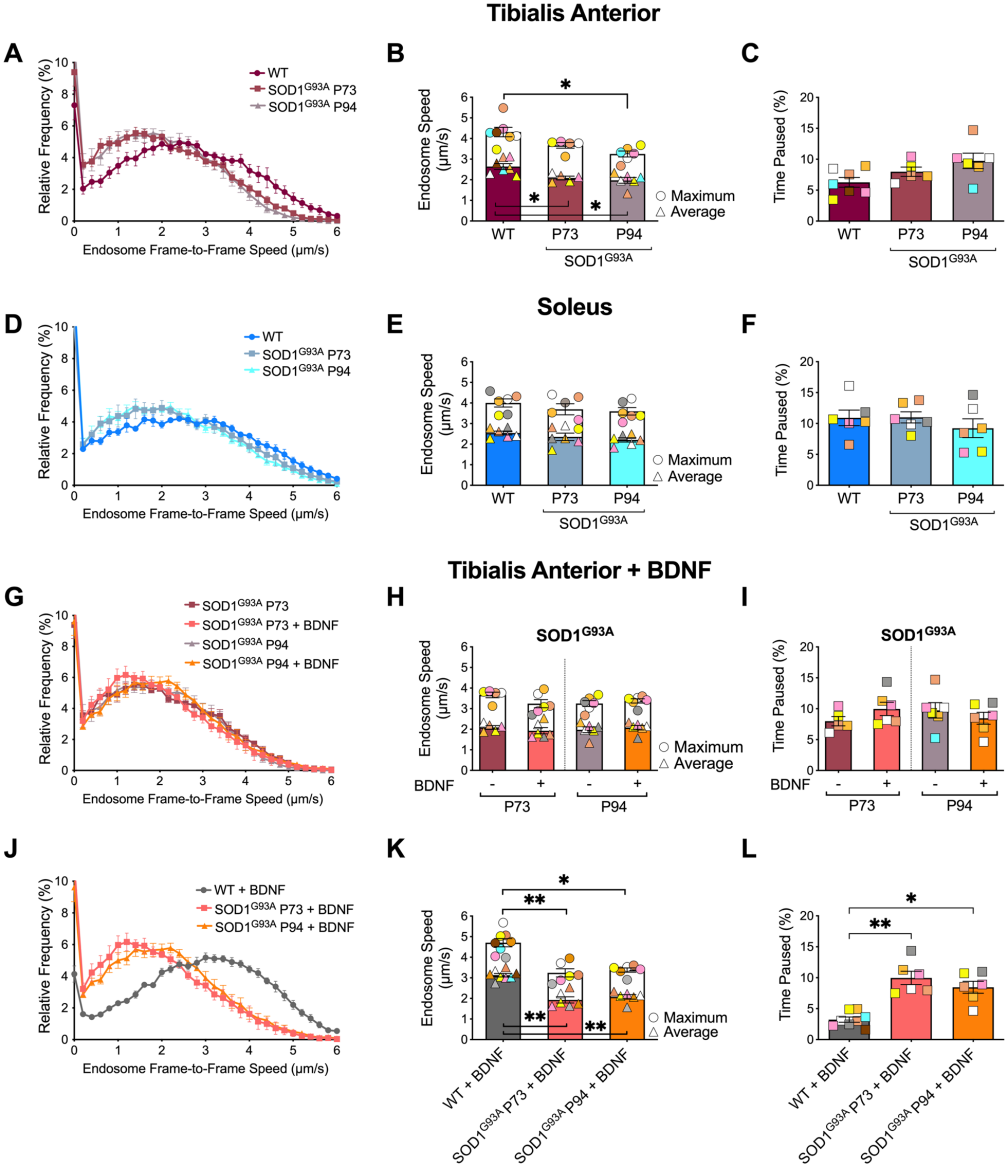
Retrograde transport and BDNF response are selectively impaired in tibialis anterior (TA) motor axons in SOD1^G93A^ mice. Retrograde transport in motor axons innervating TA in early (P73) and symptomatic (P94) SOD1^G93A^ mice compared to wild type (WT) mice, displaying: **A** endosome frame-to-frame speed distribution curves, **B** average and maximum endosome speeds (average: **p = 0.004; maximum: **p = 0.001; one-way ANOVA, n = 5–7), and the **C)** relative percentage of time signalling endosomes paused in TA-innervating axons (*p =* 0.061; one-way ANOVA, n = 5–7). Retrograde axonal transport in motor axons innervating soleus in early and symptomatic SOD1^G93A^ mice compared to WT mice displaying: **D** endosome frame-to-frame speed distribution curves, **E** average and maximum endosome speeds (average: *p =* 0.28; maximum: *p =* 0.326; one-way ANOVA, n = 5–7), and **F** relative percentage of time signalling endosomes paused in soleus-innervating axons (*p =* 0.562; one-way ANOVA, n = 6). Axonal endosome transport in P73 and P94 SOD1^G93A^ TA-innervating axons with and without BDNF stimulation, displaying: **G** endosome frame-to-frame speed distribution curves, **H** average and maximum endosome speeds (average: *p =* 0.464; maximum: *p =* 0.102; one-way ANOVA, n = 6) and **I** relative percentage of time signalling endosomes paused in TA-innervating axons with or without intramuscular BDNF stimulation (*p =* 0.521, one-way ANOVA, n = 6). Axonal endosome transport in P73 and P94 SOD1.^G93A^ vs. WT mice upon intramuscular BDNF application in motor axons innervating TA, displaying: **J** endosome frame-to-frame speed distribution curves, **K** average and maximum endosome speeds (average: ***p < 0.001; maximum: ***p < 0.001; one-way ANOVA, n = 6–8), and **L** relative percentage of time signalling endosomes paused (***p < 0.001). Means ± SEM are plotted for all graphs. *p < 0.05, **p < 0.01, Kruskal–Wallis multiple comparisons test. See also Additional file 2: Figs. S3 and S4 and Table S1

Next, we assessed in vivo axonal transport dynamics at postnatal day 73 (P73) and 94 (P94), which correspond to SOD1^G93A^ disease timepoints with ~ 20% and ~ 40% loss of lumbar MNs, respectively [5]. Axonal transport of signalling endosomes was impaired at both timepoints in SOD1^G93A^ FMNs innervating the TA (Fig. 4A, B; Additional file 2: Fig S3), and without significant alterations in pausing (Fig. 4C). Contrastingly, axonal transport was unaffected in the predominantly SMNs innervating the soleus (Fig. 4D–F; Additional file 2: Fig. S3) and the mixed population of FMNs and SMNs innervating the LG (Additional file 2: Fig. S4A–C) at both disease timepoints in SOD1^G93A^ mice.

We then assessed diameters of the axons in which endosomes were tracked to determine if there were any changes that might contribute to the transport phenotypes. Using our established methods [9], we show that the mean diameter of motor axons innervating the TA, LG and soleus are similar in WT mice (Additional file 2: Fig. S5A, B). In agreement with previous reports that TA motor units are preferentially vulnerable in ALS mice [13], we found a reduction in the mean diameters of TA motor axons at P73 (Additional file 2: Fig. S5C) that persisted and plateaued by P94 (Additional file 2: Fig. S5D). Suggestive of delayed pathology, LG motor axons displayed diameter reductions at P94 only (Additional file 2: Fig. S5D). Consistent with our in vivo axonal transport data, soleus motor axon diameters remained unaltered at both timepoints (Additional file 2: Fig. S5C, D).

We then assessed the impact of BDNF stimulation on axonal transport in SOD1^G93A^ mice (see Additional file 2: Table S1). In contrast to WT mice (e.g., Fig. 2), BDNF failed to enhance transport in SOD1^G93A^ motor axons innervating the TA (Fig. 4G–I) and LG (Additional file 2: Fig. S4D, E), whereas soleus-innervating motor axons remained unresponsive (Additional file 2: Fig. S4F, G), as also observed in WT mice. Such insensitivity to BDNF stimulation is most striking when comparing TA-innervating motor axons stimulated with BDNF in WT versus SOD1^G93A^ mice (Fig. 4J–L).

Collectively, these data demonstrate MN subtype-specific alterations in transport of signalling endosomes in SOD1^G93A^ mice and a preferential reduction of FMN axon diameters in pathology. Furthermore, diseased FMNs innervating TA become insensitive to BDNF stimulation at early symptomatic stages of ALS progression in SOD1^G93A^ mice.

### Truncated TrkB and p75^NTR^ are increased in SOD1^G93A^ muscles, but not at NMJs

**Fig. 5:**
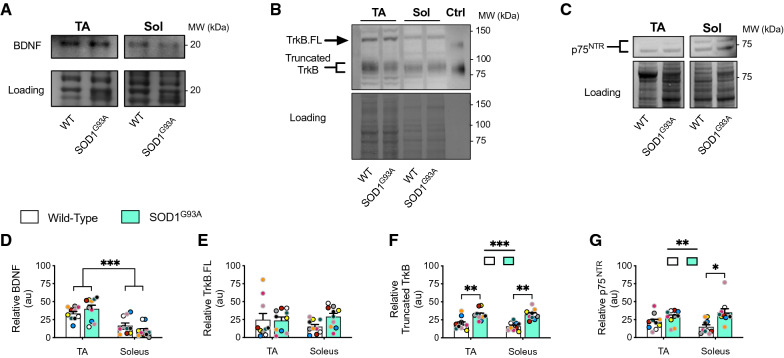
Truncated TrkB and p75^NTR^ levels are increased in SOD1^G93A^ muscles. Representative immunoblots for **A** BDNF, **B** TrkB.FL and truncated TrkB, and **C** p75^NTR^, comparing wild-type (WT) and SOD1^G93A^ tibialis anterior (TA) and soleus (Sol) muscles. Control tissue (Ctrl) from age-matched WT spinal cord (P94) was included. The difference in migration patterns suggests that TrkB.FL, but not truncated TrkB, undergoes distinct post-translational modifications in skeletal muscles. Immunoblot quantifications: **D** BDNF (genotype *p =* 0.95, muscle ***p < 0.001, interaction *p =* 0.08); **E** TrkB.FL (genotype *p =* 0.476, muscle *p =* 0.342, interaction *p =* 0.507); **F** truncated TrkB (genotype ***p < 0.001, muscle *p =* 0.239, interaction *p =* 0.384); and **G** p75^NTR^ (genotype **p = 0.008, muscle *p =* 0.932, interaction *p =* 0.14) (n = 10). Data were compared by two-way ANOVA and Holm-Šídák's multiple comparisons tests. *p < 0.05, **p < 0.01, ***p < 0.001. Means ± SEM are plotted for all graphs. Black (P73) and grey (P94) circle borders indicate age-matched mice

For subsequent experiments, we focused solely on TA and soleus muscles, because of the clear differences in axonal transport phenotypes in motor axons innervating these muscles (i.e., Fig. 4; Additional file 2: Fig. S4A–C), and their distinct fibre type compositions (i.e., TA = fast muscle; soleus = slow muscle) [11]. We first determined the levels of BDNF and its receptors in TA and soleus muscles using western blot (Fig. 5A–C). We found higher basal BDNF levels in TA compared to soleus, without significant changes in disease (Fig. 5D). Between TA and soleus muscles, there were no discernible differences in TrkB.FL (Fig. 5E); however, truncated TrkB (Fig. 5F) and p75^NTR^ (Fig. 5G) were upregulated in SOD1^G93A^ muscles.

**Fig. 6:**
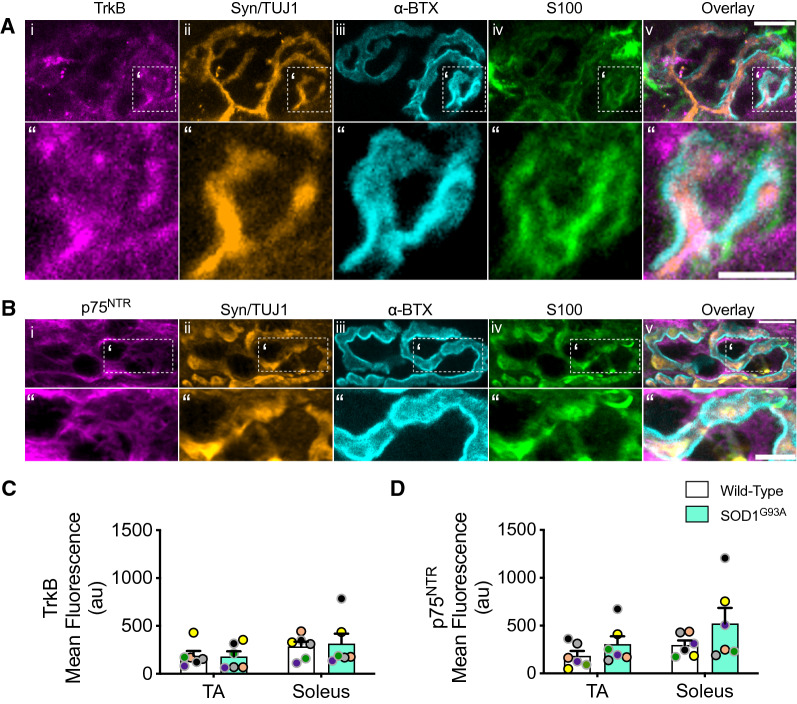
TrkB and p75^NTR^ levels are comparable at the NMJ between tibialis anterior (TA) and soleus muscles, and are not altered in disease. NMJ immunostainings of **A** (i) TrkB and **B** (i) p75^NTR^ receptors at the pre- and post-synaptic compartments as well as in terminal Schwann cells as identified by ii) Syn/TUJ1, iii) ⍺-BTX, and iv) S100 staining, respectively. The boxed area in **A-Bi-v ‘** is enlarged in **A-Bi-v“**. Scale bars in v' = 10 µm, scale bars in v" = 5 µm. Immunostaining quantifications of: **C** TrkB (genotype *p =* 0.868, muscle *p =* 0.109, interaction *p =* 0.781); and **D** p75.^NTR^ (genotype *p =* 0.091, muscle *p =* 0.108, interaction *p =* 0.603) (n = 6). Data were compared by two-way ANOVA and Holm-Šídák's multiple comparisons tests. Means ± SEM are plotted for all graphs. Black (P73) and grey (P94) circle borders indicate age-matched mice. See also Additional file 2: Fig. S6

To determine if the observed changes in neurotrophin receptors were confined to the synapse, we evaluated the synaptic expression of total TrkB and p75^NTR^ at the NMJ by immunostaining. Pre-synaptic axon terminals were identified by combined synaptophysin (Syn) and βIII-tubulin (TUJ1) staining, the post-synaptic region was labelled with ⍺-bungarotoxin (⍺-BTX), whereas terminal Schwann cells were stained with an S100 antibody. TrkB (Fig. 6A) and p75^NTR^ (Fig. 6B) staining was observed at the pre-synaptic axon terminals (i.e., Syn/TUJ1 regions), post-synaptic NMJ compartment (i.e., α-BTX regions), and peri-synaptically in terminal Schwann cells (i.e., S100 regions). By applying a Syn/TUJ1-BTX mask to the TrkB or p75^NTR^ immunolabelled regions in partially or fully innervated, but not vacant, NMJs, we found that the mean fluorescence of TrkB (Fig. 6C; Additional file 2: Fig. S6A–C) and p75^NTR^ (Fig. 6D; Additional file 2: Fig. S6D-F) did not significantly differ between TA or soleus NMJs in WT or SOD1^G93A^ mice.

Altogether, these data reveal that, whereas truncated TrkB and p75^NTR^ are increased in SOD1^G93A^ whole muscles, this increase is not reflected at the NMJ.

### TrkB.T1 and p75^NTR^ are elevated in SOD1^G93A^ sciatic nerves

**Fig. 7:**
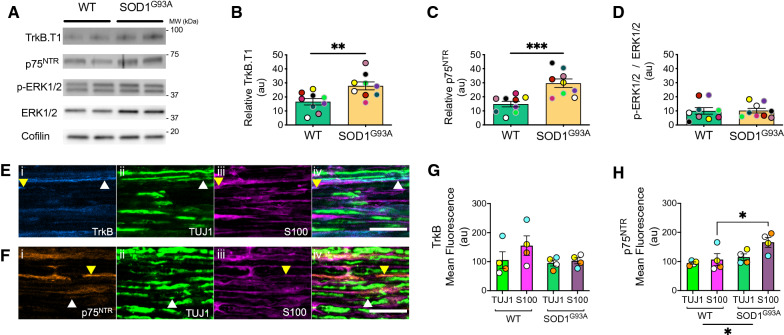
TrkB.T1 and p75^NTR^ are increased in SOD1^G93A^ sciatic nerves. **A** Immunoblots of TrkB.T1, p75^NTR^, p-ERK1/2, ERK1/2, and cofilin in WT and SOD1^G93A^ sciatic nerves. *N.B.* TrkB.FL levels were below detection. Quantification: **B** TrkB.T1 (**p = 0.007); **C)** p75^NTR^ (***p < 0.001); and **D** p-ERK1/2-ERK1/2 ratio (*p =* 0.948), by unpaired, two-tailed *t*-tests (n = 10). Black (P73) and grey (P94) circles indicate age-matched mice. Immunostaining of: **E** TrkB; and **F** p75^NTR^ in axons (TUJ1) and Schwann cells (S100). White and yellow arrowheads identify TrkB/p75^NTR^ staining in TUJ1 and S100 regions, respectively. Scale bar = 25 µm. Immunostaining quantification: **G** TrkB (genotype *p =* 0.204, cell type *p =* 0.239, interaction *p =* 0.384); and **H** p75.^NTR^ (genotype *p = 0.018, cell type *p =* 0.053, interaction *p =* 0.204). Levels in axons or Schwann cells, as assessed by two-way ANOVA and Holm-Šídák's multiple comparisons tests (n = 4). Means ± SEM are plotted for all graphs. *p < 0.05, **p < 0.01, ***p < 0.001

We then performed western blot analyses on whole sciatic nerves, probing for TrkB and p75^NTR^, as well as the phosphorylation of key downstream signalling molecules, ERK1/2 (Fig. 7A). Consistent with our muscle data (Fig. 5F-G), there is more TrkB.T1 (Fig. 7B) and p75^NTR^ (Fig. 7C) in SOD1^G93A^ sciatic nerves. However, we did not find alterations in phosphorylated ERK1/2 (Fig. 7D), confirming previous reports [7]. However, we were unable to detect TrkB.FL in our experimental conditions.

To pinpoint the cellular source of increased TrkB.T1 and p75^NTR^, we immunostained sciatic nerve sections for TrkB (Fig. 7E) and p75^NTR^ (Fig. 7F), using TUJ1 and S100 as markers of axons and Schwann cells, respectively. We then applied TUJ1 and S100 masks to TrkB (Fig. 7E) and p75^NTR^ (Fig. 7F) immunolabelled regions in WT and SOD1^G93A^ sciatic nerves. These analyses revealed no differences in TrkB content in axons or Schwann cells in WT and SOD1^G93A^ sciatic nerves (Fig. 7G). In contrast, we observed increased p75^NTR^ mean fluorescence, specifically in Schwann cells (Fig. 7H).

Collectively, these experiments further confirm that non-cell autonomous dysregulation of TrkB.T1 and p75^NTR^ signalling in peripheral sciatic nerves might contribute to SOD1^G93A^ pathology.

## Discussion

**Fig. 8:**
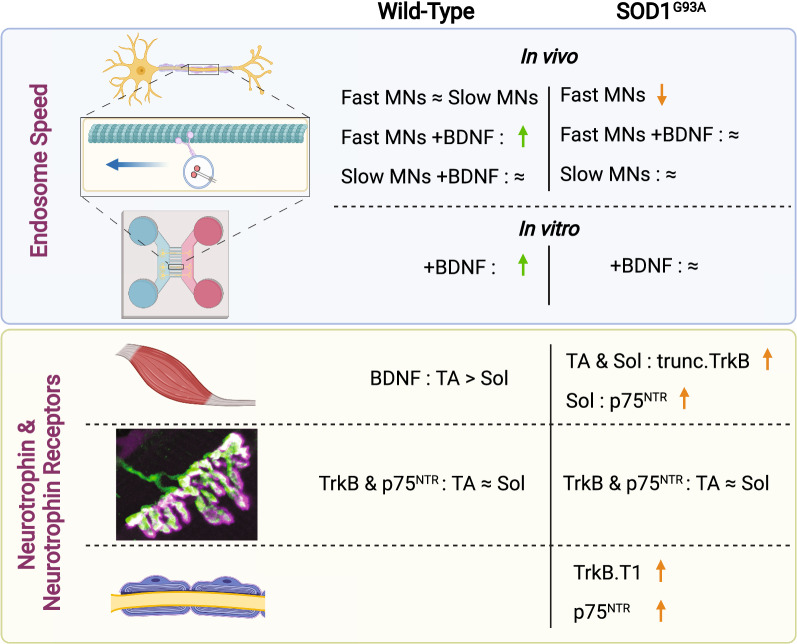
Schematic summarising the key findings from this study. In wild-type (WT) mice, retrograde signalling endosome speeds are similar between fast motor neurons (MNs) and slow MNs. BDNF stimulation increases signalling endosome speeds only in fast MNs. In SOD1^G93A^ mice, fast MNs display deficits in axonal transport and are refractory to BDNF stimulation, whereas axonal transport is unperturbed in slow MNs. Refractiveness to BDNF stimulation is also found in ventral horn spinal cord cultures isolated from SOD1^G93A^ mice. In adult WT mice, BDNF levels are higher in tibialis anterior (TA) than soleus muscles, and TrkB and p75^NTR^ levels are equivalent at the neuromuscular junction (NMJ). However, truncated TrkB and p75^NTR^ levels are increased in SOD1^G93A^ muscles and sciatic nerves, but not at the NMJ or in primary ventral horn neurons. Green upward arrows indicate an enhancement, whereas orange downward arrows highlight a negative effect. The symbol ≈ indicates no difference. Figure created with BioRender (https://biorender.com)

As summarised in Fig. 8, in this study we show that FMN and SMN axons have similar transport kinetics of signalling endosomes under basal conditions in vivo, with peripheral BDNF able to boost axonal transport speeds exclusively in FMNs. Furthermore, BDNF stimulation increases endosome speeds in WT, but not, SOD1^G93A^ primary embryonic ventral horn neuronal cultures. In early symptomatic SOD1^G93A^ mice, axonal transport is selectively impaired in FMNs innervating the TA, which also become refractory to BDNF stimulation. Moreover, pathology increases truncated TrkB and p75^NTR^ in both muscle and sciatic nerve, including in myelinating Schwann cells, but not at the NMJ. Altogether, these data suggest that cell- and non-cell autonomous BDNF signalling is impaired in an α-MN subtype-specific manner in SOD1^G93A^ pathology.

### α-MN subtypes display distinct axonal transport dynamics in WT and SOD1^G93A^ mice

This is the first study to dissect axonal transport dynamics in different α-MN subtypes in vivo. In WT mice, we found no difference in the mean or maximum endosome speeds suggesting that motor unit type does not influence basal transport dynamics. However, we observed that signalling endosomes paused less frequently in TA-innervating axons. Stationary organelles may sterically hinder axonal transport [62], forcing approaching cargoes to switch to a different microtubule track [63] to overcome obstacles on the original microtubule [64].

In basal conditions, WT and SOD1^G93A^ primary ventral horn cultures had similar axonal transport dynamics. However, application of BDNF increased axonal transport speeds in WT, but not SOD1^G93A^ primary neurons. This was not due to overt differences in TrkB.FL, truncated TrkB or p75^NTR^ receptors. Moreover, the levels of two downstream effectors of BDNF-TrkB signalling, ERK1/2 and AKT, are unchanged between WT and SOD1^G93A^ cultures [8]. However, an important caveat of these analyses is that these mixed cultures contain several neuronal (e.g., α- and γ-MN, as well as cholinergic glutamatergic, glycinergic and GABA-ergic interneurons) and non-neuronal (e.g., different glia and fibroblasts) subtypes. Furthermore, the inability of SOD1^G93A^ motor neurons to respond to BDNF may be due to multiple mechanisms, including differential recruitment of dynein adapters (e.g., snapin [65]), as well as their altered local translation [3, 42, 66]. In this regard, we have previously reported that pharmacological inhibition of IGF1R specifically increases the levels of the dynein adaptor BICD1 by promoting its axonal translation [7], thus restoring physiological transport in SOD1^G93A^ MNs in vivo.

BDNF stimulation specifically enhances axonal transport in WT FMNs, an effect not observed with GDNF. This was surprising because GDNF is important for MN survival and development [12, 30], is added to primary MN media [7–9], and enhances axonal transport of signalling endosomes in primary WT ventral horn neurons [8]. However, our observation that GDNF does not modulate axonal transport of signalling endosomes in vivo in WT FMNs, supports previous findings that specific neurotrophic factors elicit discrete signalling in different MN subtypes [22, 30, 31]. In this regard, γ-MNs require muscle spindle-derived GDNF for postnatal survival [32]. Moreover, we have recently reported that RET inhibition rescues in vivo deficits in axonal transport of signalling endosome in SOD1^G93A^ mice [8]. Altogether, this evidence indicates that neurotrophic factors elicit distinct effects on axonal transport in vivo.

We have previously demonstrated that axonal transport is impaired in pre-symptomatic SOD1^G93A^ [5–7] and TDP-43^M337V^ mice [9]. Importantly, compromised axonal transport is not a general disease by-product as heterozygous mutant FUS [9] and Kennedy’s disease [67] mice do not display in vivo transport deficits, despite displaying MN loss. However, in these studies axonal transport was assessed upon injection of both the TA and LG with BDNF. In this work, we found that only TA axons display transport deficits in SOD1^G93A^ mice and without changes in pausing, suggesting that this is not due to a general impairment in the retrograde transport machinery. Interestingly, the transport deficits observed in TA did not worsen during disease, indicative of a pathological plateau, which was also observed in TDP-43^M337V^ mice [9]. However, we are unable to account for transport dynamics in denervated MNs, as only axons with internalised H_C_T can be assessed by intravital imaging.

The precise mechanism by which FMNs selectively display impairments in retrograde axonal transport remains elusive. Mutant, but not WT, SOD1 (i.e., SOD1^G93A^ and SOD1^G85R^) interacts with the dynein motor complex [68], suggesting that vulnerable FMNs may accumulate more of this pathological protein, thus impinging upon retrograde transport regulation. Mutant SOD1 also aberrantly interacts with the stress granule protein G3BP1 [69], thus potentially disturbing processes involved in axonal maintenance (e.g., stress granule dynamics, RNA localisation) [3]. Axonal transport deficits also impact local translation, as Rab7-containing organelles, which include signalling endosomes, are sites for mitochondrial-associated local mRNA translation [66]. Whether these pathological phenomena occur specifically in vulnerable FMNs, but not in resistant SMNs, or whether other organelles also display transport deficits specifically in vulnerable FMNs, remains to be determined.

### Dysregulated truncated TrkB and p75^NTR^ in ALS mice

Dynamic NMJ remodelling precedes motor unit loss in ALS mice [17, 70], however it is currently not known whether neuromuscular BDNF signalling in fast versus slow muscles is altered in disease. Here, we report that BDNF signalling in SOD1^G93A^ mice is dysregulated in embryonic ventral horn neurons and that adult MNs are refractory to BDNF stimulation, with TA axons displaying ~ 38% reduction in transport speeds in early symptomatic SOD1^G93A^ mice (P73). As physiological BDNF and TrkB levels fluctuate (e.g., upon exercise [38]), persistent BDNF insensitivity can have severe consequences for MN homeostasis, impacting translation and signalling events in axon terminals, along the axon and within MN soma [3, 22, 38, 41, 42].

We initially hypothesised that this BDNF insensitivity might be due to: (1) reduced muscle BDNF; (2) altered TrkB and p75^NTR^ relative levels; (3) imbalanced TrkB.FL and truncated TrkB ratios; or (4) a combination of the above. In our study, we observed an increase of truncated TrkB and p75^NTR^ levels in TA and soleus muscles and sciatic nerves, suggestive of a role for these receptors in SOD1^G93A^ pathology. Remarkably, TA, which is refractory to exogenous BDNF application in SOD1^G93A^ mice and displays differential vulnerability in ALS, selectively expresses more truncated TrkB, but not p75^NTR^. The increased concentration of these receptors on the plasma membrane may reduce the availability of BDNF to bind TrkB.FL. Hence, an imbalanced ratio of TrkB.FL, truncated TrkB and p75^NTR^ could, in principle, diminish the pro-survival signalling of TrkB.FL [38], thus contributing to the vulnerability of the TA motor unit. However, the distribution of these receptors and BDNF in skeletal muscle are dynamic, as synaptic or muscular activity increases the bioavailability of BDNF and phosphorylated TrkB.FL, whilst decreasing TrkB.T1 [38]. Furthermore, symptomatic SOD1^G93A^ mice upregulate p75^NTR^ along with apoptotic markers in α-MNs [71], while deleting TrkB.T1 ubiquitously or specifically in astrocytes delays MN death in SOD1^G93A^ mice [72, 73]. Conversely, viral overexpression of TrkB.T1 induces MN degeneration [74]. These studies suggest that BDNF-mediated signalling pathways are altered in ALS and that their modulation might have therapeutic benefits [35]. Indeed, harnessing the pro-survival activity of p75^NTR^ prevents MN death and extends the lifespan of SOD1^G93A^ mice, in part, by rescuing p-TrkB, p-Akt, p-ERK and p-CREB levels in SOD1^G93A^ spinal cords [75].

However, a caveat to our TrkB immunostaining approach is that the commercially available TrkB antibodies bind to the extracellular domain of this receptor, and thus cannot distinguish TrkB.FL from TrkB.T1. A limitation of our experimental approach is that while western blotting allows us to evaluate the TrkB isoforms, it lacks MN subtype specificity; conversely, our immunostaining experiments enable MN subtype detection, but lack TrkB isoform differentiation. Hence, dissecting the endogenous levels of TrkB isoforms in FMNs and SMNs is currently not possible. In addition, we observed tissue-specific differences in the molecular weights of full-length and truncated TrkB, as well as p75^NTR^ receptors. In skeletal muscle and embryonic ventral horn cultures, TrkB.FL migrated at ~ 140 kDa (Fig. 5B and Additional file 2: Fig. S2ai), whereas in spinal cord and brain, we observed TrkB.FL at ~ 120 kDa (Fig. 5B and data not shown). In skeletal muscle, TrkB.T1 and TrkB.T2 were identified at ~ 80–95 kDa, whereas in sciatic nerve, the TrkB.T1 isoform was observed as a single band at ~ 95 kDa. Such differences may be due to tissue-specific post-translational modifications [76]. For example, there are ten N-terminal glycosylation sites in TrkB [77], and its phosphorylated form has been observed at both ~ 120 kDa and ~ 140 kDa [75] in WT and SOD1^G93A^ spinal cords. Total and phosphorylated TrkB.FL have also been shown to migrate at ~ 140 kDa in skeletal muscle [37]. p75^NTR^ was detected as two distinct bands at ~ 75 kDa and ~ 85 kDa in skeletal muscle (Fig. 5C), likely due to N- and O-linked glycosylation [78], with the upper band (i.e., ~ 85 kDa) representing the fully glycosylated form and the lower band (i.e., ~ 75 kDa) the non-glycosylated form [79].

Collectively, our data indicate that the BDNF signalling axis is essential for maintenance and homeostatic regulation of FMNs, which is selectively impaired in SOD1^G93A^ pathology in a cell- and non-cell autonomous manner.

## Supplementary Information

**Additional file 1**. **Video S1.** Intravital time-lapse microscopy of HCT-555-positive signalling endosomes (white) in at least three sciatic nerve axons of a live, anaesthetised mouse.

**Additional file 2**. **Figure S1.** Retrograde transport dynamics of signalling endosomes in wild-type mice; **Figure S2.** TrkB.FL, truncated TrkB and p75^NTR^ levels do not differ between wild-type (WT) and SOD1^G93A^ primary embryonic ventral horn neurons in mass culture; **Figure S3.** Kymographs of in vivo retrograde transport of HCT-555-positive signalling endosomes from live, anesthetised mice; **Figure S4.** Retrograde transport dynamics of signalling endosomes in axons innervating lateral gastrocnemius (LG) and soleus muscles in WT and SOD1^G93A^ mice; **Figure S5.** Fast motor axon diameters decrease with progression of SOD1^G93A^ pathology; **Figure S6.** TrkB and p75^NTR^ expression at the neuromuscular junction (NMJ) in WT and SOD1G93A tibialis anterior and soleus muscles; **Table S1.** Number of animals, axons, cargoes and frame-to-frame movements assessed for each in vivo axonal transport experimental group; **Table S2.** Number of animals, axons, cargoes and frame-to-frame movements assessed for each primary ventral horn culture used for in vitro axonal transport experiments; **Table S3.** Primary antibodies used in this study; **Table S4.** Secondary antibodies used in this study.

## Data Availability

All data generated or analysed during this study are included in this published article [and associated supplementary information].
