# Supplementary material for: BDNF-dependent modulation of axonal transport is selectively impaired in ALS
Source: Acta Neuropathol Commun. 2022 Aug 22;10:121. doi: 10.1186/s40478-022-01418-4 (PMC9396851; doi:10.1186/s40478-022-01418-4)
Supplement: Supplementary file 2 — Additional file 2. Figure S1. Retrograde transport dynamics of signalling endosomes in wild-type mice; Figure S2. TrkB.FL, truncated TrkB and p75NTR levels do not differ between wild-type (WT) and SOD1G93A primary embryonic ventral horn neurons in mass culture; Figure S3. Kymographs of in vivo retrograde transport of HCT-555-positive signalling endosomes from live, anesthetised mice; Figure S4. Retrograde transport dynamics of signalling endosomes in axons innervating lateral gastrocnemius (LG) and soleus muscles in WT and SOD1G93A mice; Figure S5. Fast motor axon diameters decrease with progression of SOD1G93A pathology; Figure S6. TrkB and p75NTR expression at the neuromuscular junction (NMJ) in WT and SOD1G93A tibialis anterior and soleus muscles; Table S1. Number of animals, axons, cargoes and frame-to-frame movements assessed for each in vivo axonal transport experimental group; Table S2. Number of animals, axons, cargoes and frame-to-frame movements assessed for each primary ventral horn culture used for in vitro axonal transport experiments; Table S3. Primary antibodies used in this study; Table S4. Secondary antibodies used in this study. [file 40478_2022_1418_MOESM2_ESM.pdf]

Supplementary Material for "BDNF-dependent modulation of axonal transport is selectively impaired in ALS"

Andrew P. Tosolini [1,2 *], James N. Sleigh [1,2,3 #], Sunaina Surana [1,2,3 #], Elena R. Rhymes [1,2], Stephen D. Cahalan [4] and Giampietro Schiavo [1,2,3,5*]

[1] Department of Neuromuscular Diseases, Queen Square Institute of Neurology, University College London, WC1N 3BG, United Kingdom.

[2] UCL Queen Square Motor Neuron Disease Centre, University College London, WC1N 3BG, United Kingdom.

[3] UK Dementia Research Institute, University College London, WC1E 6BT, United Kingdom.

[4] Comparative Neuromuscular Disease Laboratory, Department of Clinical Sciences and Services, Royal Veterinary College, University of London, NW1 0TU, United Kingdom.

[5] Lead contact.

[#] These authors contributed equally to this work.

[*] Correspondence to:

Andrew P. Tosolini - a.tosolini@ucl.ac.uk; Tel: +44(0)20 3448 4334

Giampietro Schiavo - giampietro.schiavo@ucl.ac.uk Tel: +44(0)20 7679 8007

Video S1 legend**, Figures S1-6** and legends**, Tables S1-4.**

Supplementary Movie

Video S1. Intravital time-lapse microscopy of H_CT-555-positive signalling endosomes (white) in at least three sciatic nerve axons of a live, anaesthetised mouse. **A)** Unprocessed time-lapse video. **B)** Using the TrackMate plugin in FIJI/ImageJ, the automated spot detection method highlights all cargoes with purple circles as determined by several parameters (*i.e.*, fluorescence intensity, diameter). **C)** Frame-to-frame manual tracking shows the selected cargoes processed for further analyses from the top and bottom axons. **D)** Overlay of all H_CT-555-positive signalling endosome tracks included for further analyses. Frame rate = 0.388 s; playback = 10 frames/s; total frames = 750; scale bar = 5 μm. Retrograde movement from right to left.

Supplementary Figures

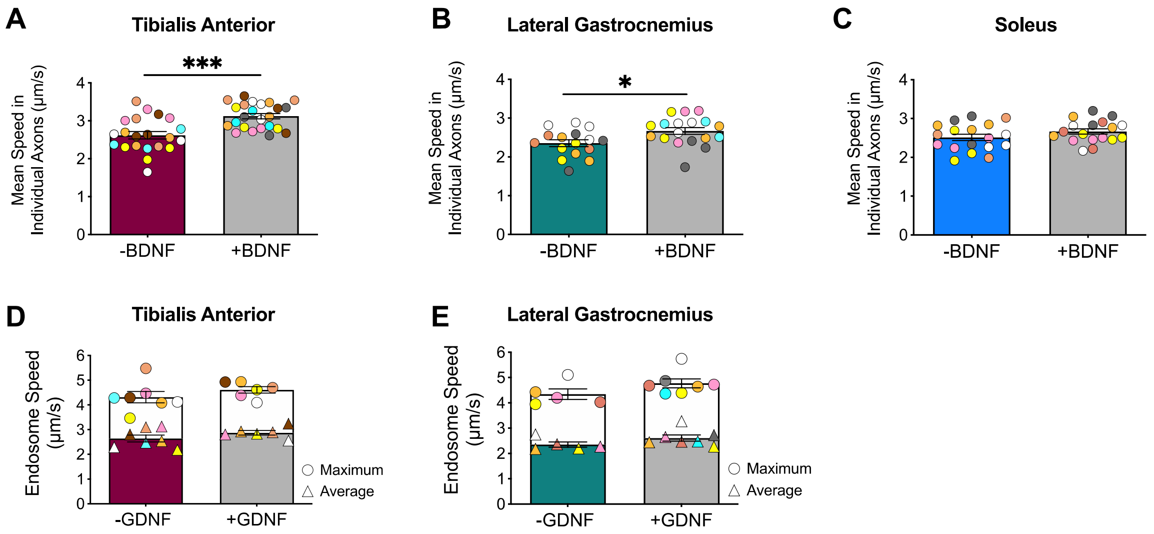

Figure S1. Retrograde transport dynamics of signalling endosomes in wild-type mice. A) Mean axonal endosome speeds with and without intramuscular BDNF stimulation of motor neurons innervating TA (*** $p < 0.001$), **B)** LG (* $p = 0.0167$), and **C)** soleus ($p = 0.161$), as assessed by two-tailed, unpaired *t*-tests (n = 15-24). The colour coding is consistent with **Fig. 2**. **D)** Average (triangles) and maximum (circles) axonal endosome speeds upon intramuscular GDNF stimulation in TA (average: $p = 0.181$; maximum: $p = 0.181$) and **E)** LG wild-type motor neurons (average: $p = 0.149$; maximum: $p = 0.149$), as assessed by a two-tailed unpaired Mann-Whitney U test (n = 6-7). Means ± SEM are plotted for all graphs. Linked to **Fig. 2**.

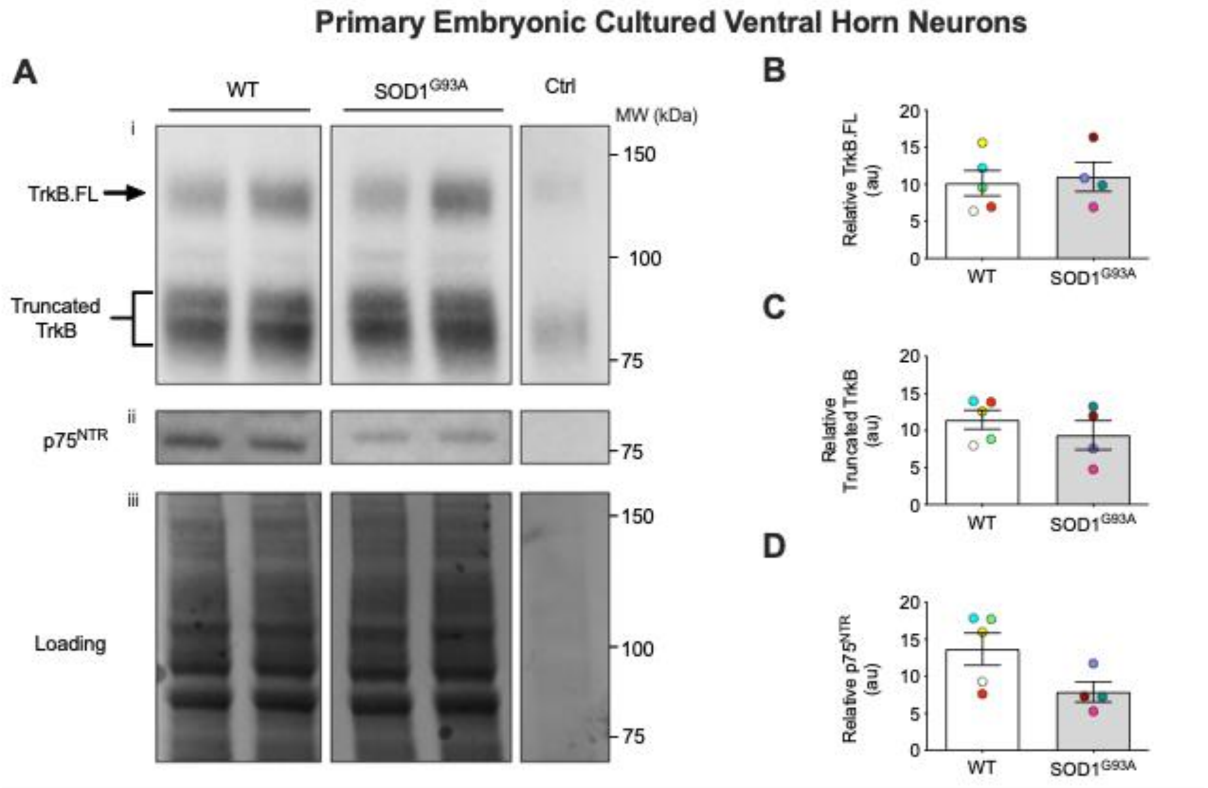

Figure S2. TrkB.FL, truncated TrkB and p75^{NTR} levels do not differ between wild-type (WT) and SOD1^{G93A} primary embryonic ventral horn neurons in mass culture. A) Representative immunoblots of TrkB.FL and truncated TrkB isoforms (i), p75^{NTR} (ii) and total protein loading control (iii). WT adult spinal cord was included as positive control tissue (Ctrl). Quantifications reveal no difference in the expression of **B)** TrkB.FL ($p = 0.732$), **C)** truncated TrkB ($p = 0.2857$), and **D)** p75^{NTR} ($p = 0.0635$). Data were assessed by a Mann-Whitney test (n = 4-5 biological replicates). Means ± SEM are plotted for all graphs. The colour coding of individual datapoints is consistent across graphs and reflects the same biological replicate.

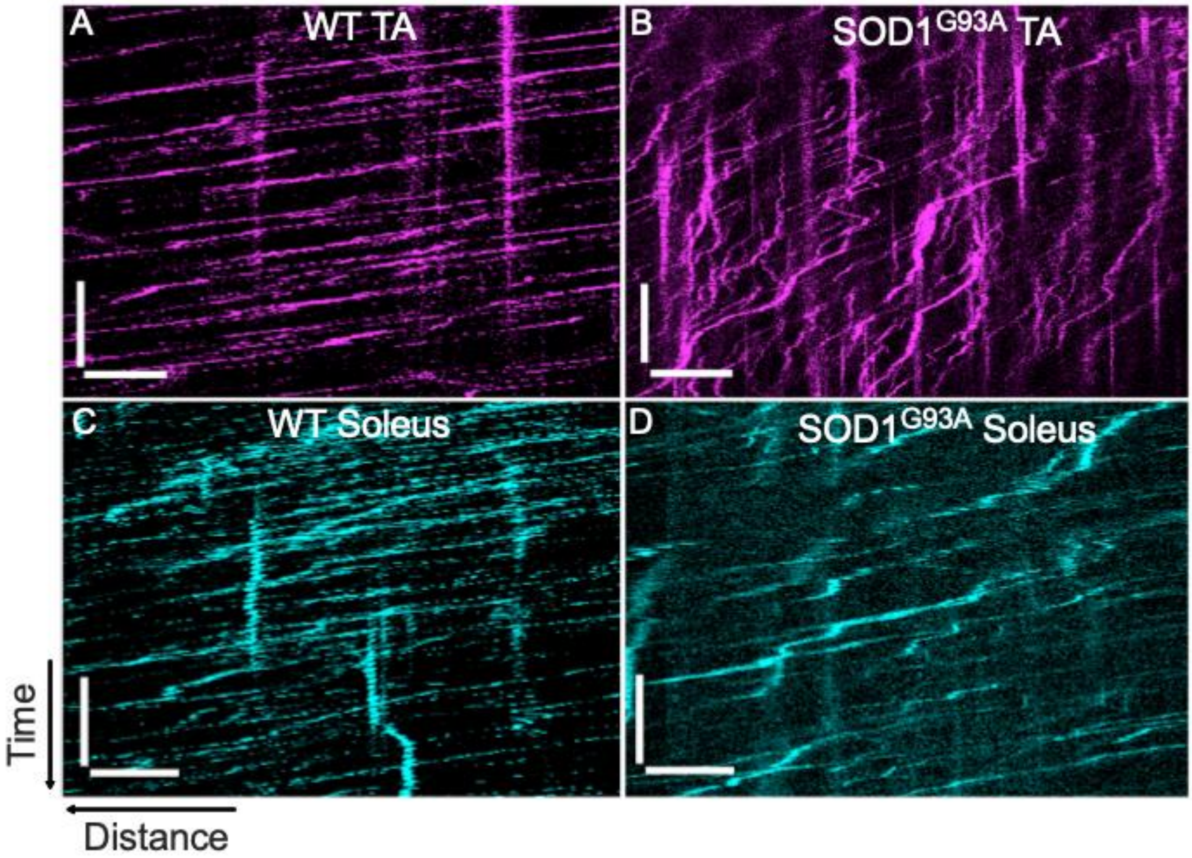

Figure S3. Kymographs of *in vivo* retrograde transport of H_CT-555-positive signalling endosomes from live, anesthetised mice. A-D) Representative kymographs of retrograde transport of signalling endosomes in motor axons innervating wild-type (WT) tibialis anterior (TA, A) and soleus (C) muscles and SOD1^{G93A} TA (B) and soleus (D) muscles. Retrogradely moving H_CT-555-positive signalling endosomes are represented by right-to-left movements and paused H_CT-555-positive signalling endosomes are represented by vertical lines. Distance (x-axis) scale bars = 10 µm; time (y-axis) scale bars = 30 s.

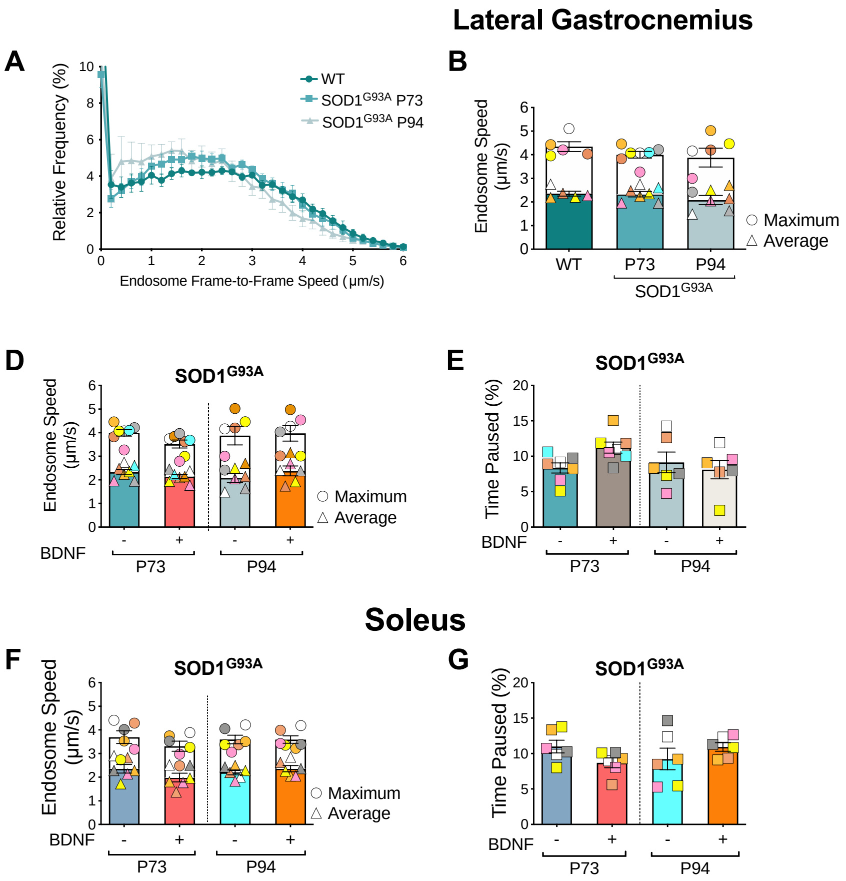

Figure S4. Retrograde transport dynamics of signalling endosomes in axons innervating lateral gastrocnemius (LG) and soleus muscles in WT and SOD1^{G93A} mice. Retrograde transport of signalling endosomes in motor axons innervating LG at two stages of SOD1^{G93A} disease (P73 and P94) compared to WT mice, displaying: **A)** endosome frame-to-frame speed distribution curves; **B)** average and maximum endosome speeds (average: $p = 0.492$; maximum: $p = 0.870$; one-way ANOVA, n = 5-7); and **C)** relative percentage of time signalling endosomes paused ($p = 0.128$; one-way ANOVA, n = 5-7). Axonal endosome transport in P73 and P94 SOD1^{G93A} LG-innervating axons with and without BDNF stimulation, displaying: **D)** average and maximum endosome speeds (average: $p = 0.384$; maximum: $p = 0.257$; one-way ANOVA, n = 6); and **E)** percentage of time signalling endosomes paused ($p = 0.136$, one-way ANOVA, n = 6). Axonal transport of signalling endosomes in P73 and P94 SOD1^{G93A} MN axons innervating the soleus with and without BDNF stimulation, displaying: **F)** average and maximum endosomal speeds (average: $p = 0.48$; maximum: $p = 0.669$; one-way ANOVA, n = 6); and **G)** relative percentage of time signalling endosomes paused ($p = 0.213$; one-way ANOVA, n = 6). Statistics were assessed by a one-way ANOVA followed by a Kruskal-Wallis multiple comparisons test. The colour coding of individual datapoints is consistent within the muscle and treatment type and reflects the same animal. Linked to **Fig. 4.**

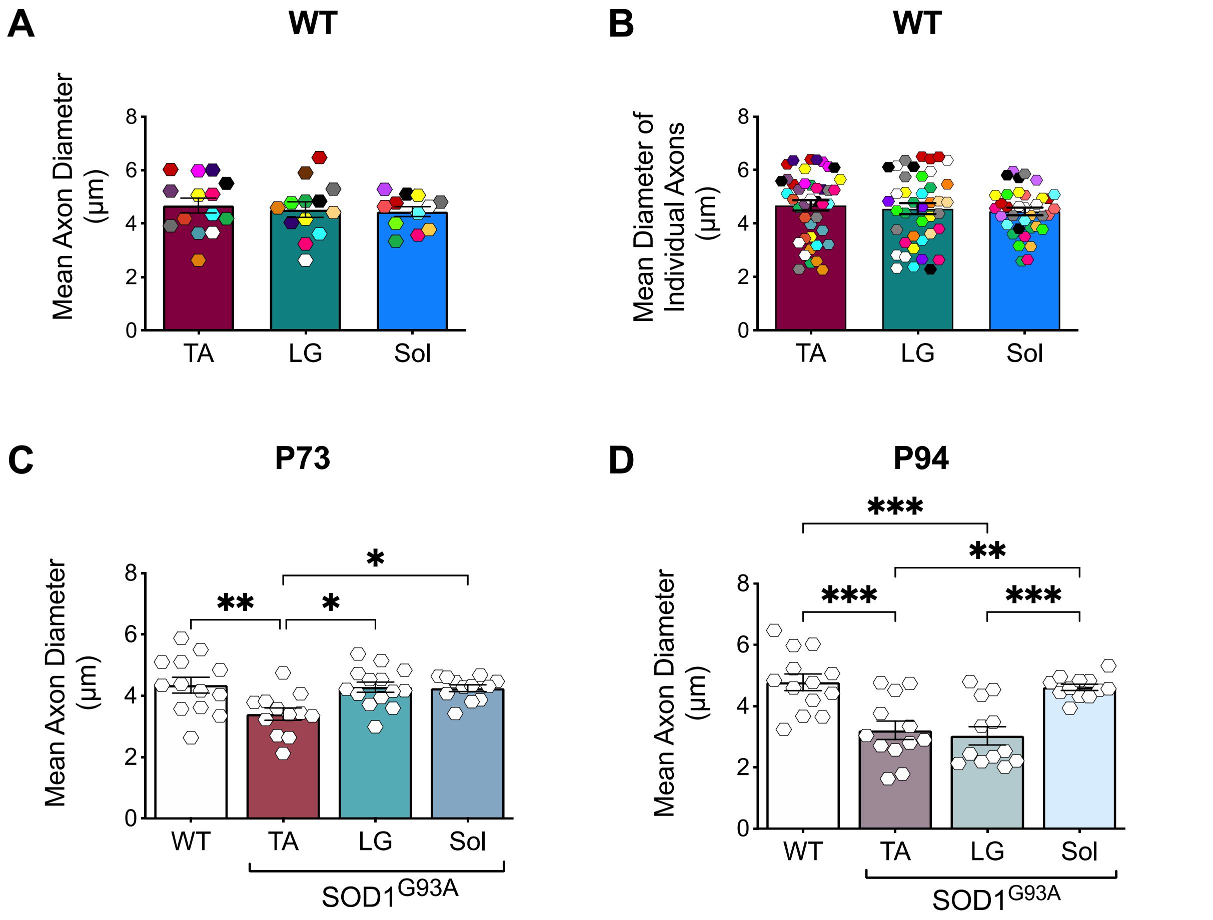

Figure S5. Fast motor axon diameters decrease with progression of SOD1^{G93A} pathology. A) Mean diameter of H$_C$T-555 containing motor axons innervating tibialis anterior (TA), lateral gastrocnemius (LG), and soleus (Sol) per mouse ($p = 0.819$, one-way ANOVA, $n = 16\text{-}21$). **B)** Mean diameters of individual motor axons innervating TA, LG and Sol that contain H$_C$T-555-positive signalling endosomes ($p = 0.695$, one-way ANOVA, $n = 36\text{-}42$). The colour coding is consistent with **Fig. S5A**. Mean diameter of H$_C$T-containing motor axons innervating TA, LG, and Sol in SOD1^{G93A} and wild-type (WT) sciatic nerves at **C)** P73 (** $p = 0.004$) and **D)** P94 (*** $p < 0.001$), as assessed by one-way ANOVA and Holm-Šídák's multiple comparisons test ($n = 12\text{-}14$). Means ± SEM are plotted for all graphs. The colour coding of individual datapoints is consistent within the muscle type and reflects the same animal in **Figs. S5A and S5B**. * $p < 0.05$, ** $p < 0.01$, *** $p < 0.001$.

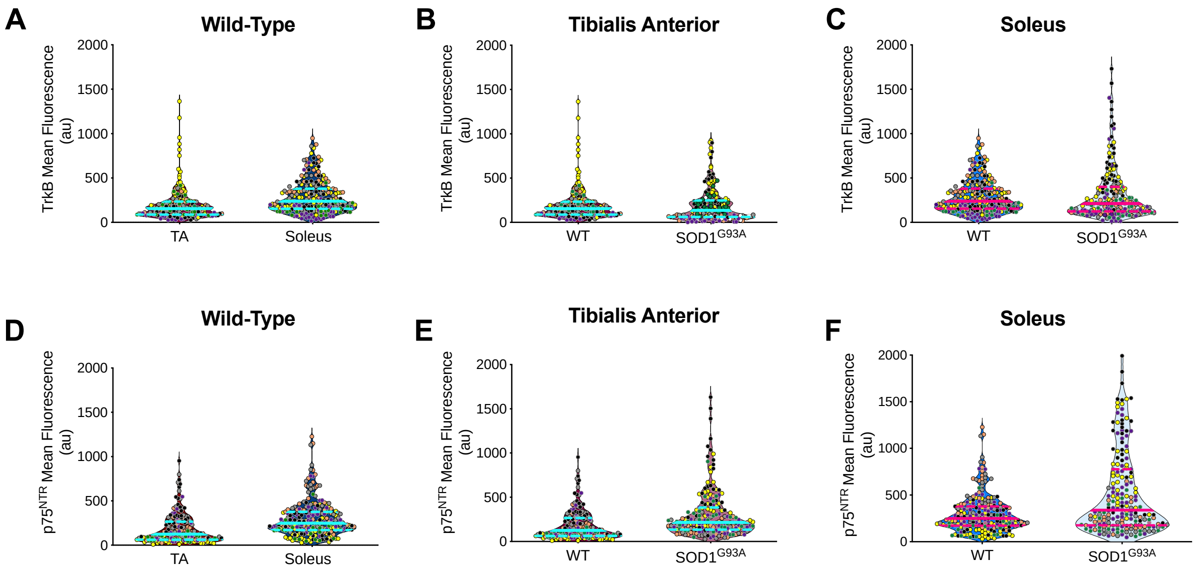

Figure S6. TrkB and p75^{NTR} expression at the neuromuscular junction (NMJ) in WT and SOD1^{G93A} tibialis anterior and soleus muscles. A) Individual mean fluorescence data points of TrkB at the NMJ in WT tibialis anterior (182) and soleus (223) muscles (n = 6). **B)** Individual mean fluorescence data points of TrkB at the NMJ in tibialis anterior muscles of WT (182 NMJs) and SOD1^{G93A} (180 NMJs) mice (n = 6). **C)** Individual mean fluorescence data points of TrkB at the NMJ in soleus muscles comparing WT (223 NMJs) and SOD1^{G93A} (191 NMJs) mice (n = 6). Panels A-C are linked to **Fig. 6C**. **D)** Individual mean fluorescence data points of p75^{NTR} at the NMJ in WT tibialis anterior (176) and soleus (224) muscles (n = 6). **E)** Individual mean fluorescence data points of p75^{NTR} at the NMJ in tibialis anterior of WT (176 NMJs) and SOD1^{G93A} (183 NMJs) mice (n = 6). **F)** Individual mean fluorescence data points of p75^{NTR} at the NMJ in soleus muscles comparing WT (224 NMJs) and SOD1^{G93A} (198 NMJs) mice (n = 6). Panel D-F are linked to **Fig. 6D**. Colour coding of individual data points represents an individual animal, and match their corresponding graph in **Figs. 6C-D**. The cyan/pink lines represent the mean, and the dashed cyan/pink lines represent the upper and lower quartiles. Black (P73) and grey (P94) circles indicate age-matched mice.

Table S1. Number of animals, axons, cargoes and frame-to-frame movements assessed for each *in vivo* axonal transport experimental group.

Muscle + stimulant	Genotype	Number of Animals	Number of Axons	Number of signalling endosomes	Number of Frame-to-Frames Movements	Mean Velocity (µm/s)	Max Velocity (µm/s)	Pausing (%)
**TA**	Wild-type	7	21	469	19,405	2.64	4.31	6.26
**TA + BDNF**	Wild-type	8	24	690	24,543	3.13	4.70	3.23
**TA + GDNF**	Wild-type	6	18	530	18,445	2.87	4.61	3.13
**TA**	$SOD1^{G93A}$ P73	5	15	269	15,508	2.09	3.66	7.98
**TA**	$SOD1^{G93A}$ P94	6	18	309	18,880	1.97	3.25	9.74
**TA + BDNF**	$SOD1^{G93A}$ P73	6	18	326	18,779	1.93	3.25	9.97
**TA + BDNF**	$SOD1^{G93A}$ P94	6	18	338	19,009	2.08	3.38	8.45
**Soleus**	Wild-type	6	18	292	18,737	2.54	4.00	10.91
**Soleus + BDNF**	Wild-type	6	18	309	18,932	2.68	4.00	10.34
**Soleus**	$SOD1^{G93A}$ P73	6	18	246	18,416	2.35	3.69	10.99
**Soleus**	$SOD1^{G93A}$ P94	6	18	238	18,574	2.20	3.60	9.23
**Soleus + BDNF**	$SOD1^{G93A}$ P73	6	18	241	18,484	1.98	3.31	8.69
**Soleus + BDNF**	$SOD1^{G93A}$ P94	6	18	254	18,493	2.36	3.59	10.93
**LG**	Wild-type	5	16	609	25,402	2.35	4.34	12.26
**LG + BDNF**	Wild-type	6	19	581	21,978	2.75	4.43	5.56
**LG + GDNF**	Wild-type	7	29	947	30,660	2.61	4.77	4.60
**LG**	$SOD1^{G93A}$ P73	7	21	430	21,517	2.32	4.00	8.34
**LG**	$SOD1^{G93A}$ P94	6	18	408	18,241	2.08	3.88	9.13
**LG + BDNF**	$SOD1^{G93A}$ P73	7	21	371	21,929	2.13	3.52	11.22
**LG + BDNF**	$SOD1^{G93A}$ P94	6	18	394	18,540	2.38	3.97	8.11

Table S2. Number of animals, axons, cargoes and frame-to-frame movements assessed for each primary ventral horn culture used for *in vitro* axonal transport experiments.

Genotype + stimulant	Number of Biological Replicates	Number of Axons	Number of signalling endosomes	Number of Frame-to-Frames Movements	Mean Velocity (µm/s)	Pausing (%)
**Wild-type**	3	24	103	12,650	1.42	26.44
**SOD1^{G93A}**	3	22	119	11,769	1.48	23.39
**Wild-type + BDNF**	3	22	289	21,747	1.74	27.96
**SOD1^{G93A} + BDNF**	3	21	238	20,007	1.43	29.47

Table S3. Primary antibodies used in this study. *CST*, Cell Signaling Technology; *IHC*, immunohistochemistry; *RRID*, research resource identifier; *WB*, western blot.

Target	Species	Clonality	Company	Catalogue #	RRID	IHC dilution	WB dilution
**α-Bungarotoxin, CF®405S**	n/a	n/a	Biotium	BT00002	n/a	1:500	n/a
**α-Bungarotoxin-594**	n/a	n/a	ThermoFisher Scientific	B13423	n/a	1:500	n/a
**ß-III Tubulin (TUJ1)**	Chicken	Poly	Synaptic Systems	302306	AB_2620048	1:500 (sciatic nerve) 1:50 (NMJ)	n/a
**BDNF**	Rabbit	Poly	Alomone	ANT-010	AB_2039756	n/a	1:1,000 (muscle)
**Cofilin**	Rabbit	Poly	Cytoskeleton	ACFL02	AB_10708808	n/a	1:500 (sciatic nerve)
**ERK1/2**	Rabbit	Poly	CST	9102	AB_330744	n/a	1:1,000 (sciatic nerve)
**pERK1/2 (T202/T204)**	Rabbit	Poly	CST	9101	AB_331646	n/a	1:500 (sciatic nerve)
**$p75^{NTR}$**	Rabbit	Poly	Biolegend (Covance)	Poly18397 (PRB-602C)	AB_2565441	1:500 (primary neurons)	1:1,000 (muscle) 1:2,000 (sciatic nerve)
**$p75^{NTR}$**	Rabbit	Poly	Promega	G3231	AB_430853	1:50 (NMJ) 1:500 (sciatic nerve)	n/a

**S100**	Mouse	Mono	Merck	S2532	AB_477499	1:200 (sciatic nerve)	n/a
**S100**	Mouse	Mono	Atlas Antibodies	AMAb91038	AB_2665776	1:250 (NMJ)	n/a
**Synaptophysin**	Chicken	Poly	Synaptic Systems	101-006	AB_2622239	1:50 (NMJ)	n/a
**TrkB**	Rabbit	Poly	Millipore	07-225	AB_310445	1:50 (NMJ) 1:250 (sciatic nerve)	1:1,000 (muscle) 1:1,000 (sciatic nerve)
**TrkB**	Goat	Poly	R&D Systems	AF1494	AB_2155264	1:500 (primary neurons)	1:1,000 (muscle)

Table S4. Secondary antibodies used in this study. *HRP*, horseradish peroxidase; *RRID*, research resource identifier.

**Target**	**Species**	**Conjugate**	**Company**	**Catalogue #**	**RRID**	**Dilution**
**Chicken IgY**	Goat	Alexa Fluor 555	ThermoFisher Scientific	A-21437	AB_2535858	1:500 (sciatic nerve)
**Chicken IgY**	Goat	Alexa Fluor 647	ThermoFisher Scientific	A-21449	AB_2535866	1:200 (NMJ)
**Mouse IgG**	Goat	Alexa Fluor 647	ThermoFisher Scientific	A-21236	AB_2535805	1:500 (sciatic nerve)
**Rabbit IgG**	Goat	Alexa Fluor 488	ThermoFisher Scientific	A-11034	AB_2576217	1:500 (sciatic nerve) 1:200 (NMJ)
**Rabbit Ig**	Donkey	HRP	Bio-Rad	1706515	AB_11125142	1:3,000 (sciatic nerve)
**Rabbit IgG**	Mouse	HRP	Jackson Laboratories	211-032-171-JIR	AB_2339149	1:1,000 (muscle)
**Rabbit TrueBlot®: Anti-Rabbit IgG**	Mouse	HRP	Rockland Immunochemicals	18-8816-31	AB_2610847	1:1,000 (muscle)
**Goat Ig**	Rabbit	HRP	DAKO (Agilent)	P0449	AB_2617143	1:1,000 (primary neurons, muscle)
**Mouse IgG**	Rabbit	HRP	DAKO (Agilent)	Z0412	AB_2810286	1:1,000 (primary neurons, muscle)
